# Pulvinar pathways as skip connections in deep neural networks for vision

**DOI:** 10.3389/fnimg.2026.1800369

**Published:** 2026-05-14

**Authors:** Narmin Zarinabadi, Christian Casanova, Nelson Cortes

**Affiliations:** Visual Neuroscience Laboratory, School of Optometry, Université de Montréal, Montreal, QC, Canada

**Keywords:** contrast-dependent gain control, ignition threshold regulation, near-threshold decision criterion, Pulvinar, representation stabilization, skip-connections, transthalamic connections, decision weighting

## Abstract

**Introduction:**

The pulvinar is thought to regulate cortico-cortical communication through transthalamic pathways, yet the computational consequences of such modulation remain unclear.

**Methods:**

A minimal pulvinar-inspired long-range skip pathway in a hierarchical convolutional vision model was tested, implemented as a learned projection from early to late feature maps coupled with a gain-controlled gating mechanism. The architecture was evaluated in two complementary experiments: CIFAR-10 categorization and a near-threshold contrast-detection task with noisy backgrounds.

**Results:**

In CIFAR-10 categorization, the pulvinar-augmented model did not primarily act as a uniform accuracy booster; instead, it reshaped contrast-dependent response scaling and stabilized internal representations, consistent with gain control. These effects persisted under matched representational regimes, indicating that performance differences cannot be explained by changes in representational geometry alone. In the near-threshold detection task, pulvinar-like modulation systematically redistributed detection outcomes, consistent with shifts in decision criterion under uncertainty, and in specific regimes supported improved discriminability.

**Discussion:**

Together, these findings indicate that pulvinar-inspired connectivity contributes primarily to gain and decision-criterion control. We propose that the pulvinar regulates how sensory evidence is weighted and converted into decisions, rather than directly enhancing task accuracy.

## 1 Introduction

The pulvinar has become increasingly central to contemporary theories of thalamocortical computation. Rather than serving as a passive relay of sensory information, as classical models proposed, the pulvinar is now understood as an active regulatory hub that shapes cortical communication, attention, and hierarchical integration (Cortes et al., 2024, 2026). Extensive anatomical studies demonstrate that the pulvinar receives dense, reciprocal projections from nearly all levels of the visual cortical hierarchy as well as convergent input from the superior colliculus, placing it at a critical intersection of subcortical and cortical pathways (Casanova and Chalupa, 2023). Physiological evidence further reveals that pulvinar neurons exhibit receptive-field properties comparable to cortical neurons, including selectivity for orientation, direction of motion, disparity, and global motion patterns (Casanova et al., 2001). These properties are strongly dependent on cortical inputs: inactivation of V1 sharply reduces responses in pulvinar subdivisions such as the striate-recipient zone, demonstrating that cortical feedback is essential for shaping pulvinar activity (Casanova and Darveau, 1997).

Beyond relaying visual signals, the pulvinar exerts widespread influences on cortical processing. Pulvinar projections can alter firing rates, shift response gain, and reshape spatial and temporal response profiles in multiple cortical regions including V1, V2, V4, and high-level temporal cortex (Casanova and Chalupa, 2023; Casanova, 2020; Sherman, 2017). A key mechanism underlying this influence is the pulvinar's ability to regulate effective connectivity between cortical areas (Cortes et al., 2024), dynamically modulating the strength of cortico-cortical interactions, effectively determining how strongly one cortical population drives another (Shipp, 2003; Saalmann et al., 2012). This modulation is highly context dependent: pulvinar activity selectively enhances task-relevant visual information and attentional priority (Robinson and Petersen, 1992; Zhou et al., 2016), consistent with theories that the pulvinar implements precision or gain control within a predictive coding (Kanai et al., 2015; Olsen et al., 2012; Cortes et al., 2024, 2026).

The pulvinar also plays a critical role in coordinating the timing of communication across the cortical hierarchy. The pulvinar organizes interareal synchrony, influencing gamma- and theta-band coupling that regulates when different cortical populations communicate (Saalmann et al., 2012; Wróbel, 2000; Cortes et al., 2026, 2020). This ability to selectively synchronize distributed cortical regions implies that the pulvinar not only routes information but also structures the temporal windows in which communication can occur. Such temporal modulation is essential for functions such as attention, object segmentation, and visuomotor coordination (Miura and Scanziani, 2022).

**Figure 1:**
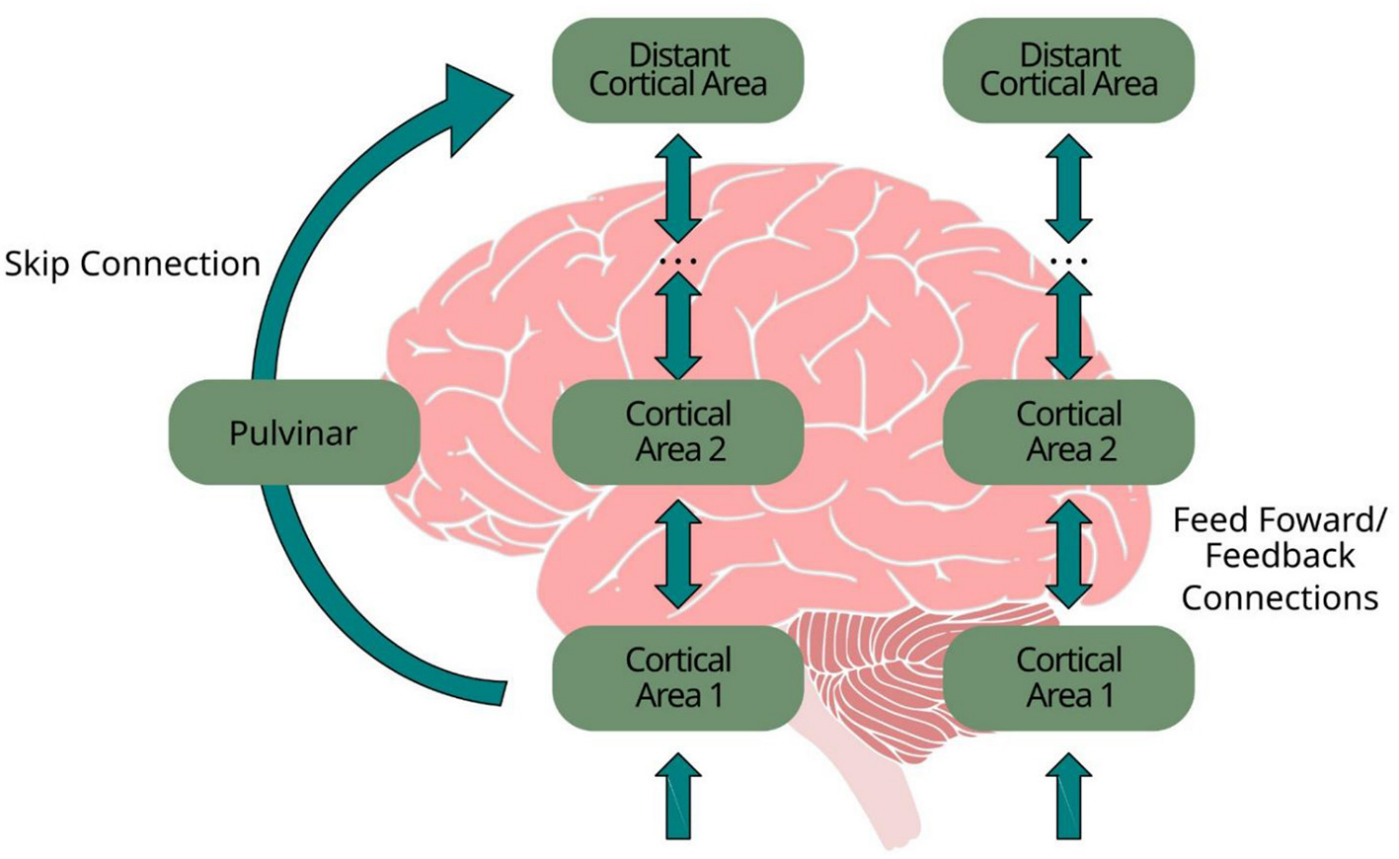
Schematic illustration of transthalamic (pulvinar-mediated) communication. Early cortical areas project to the pulvinar, which relays and modulates signals to distant cortical areas, enabling long-range, context-dependent routing that parallels skip connections in deep neural networks.

These findings have led to the modern view of the pulvinar as a transthalamic communication hub. In this framework, the pulvinar belongs to a class of higher-order thalamic (HO) nuclei, defined as thalamic structures that receive strong driving input from cortex and participate in cortico-thalmo-cortical loops rather than simply relaying peripheral sensory signals (Sherman and Guillery, 2011). Through these transthalamic pathways, HO nuclei can mediate indirect corticocortical interactions, effectively linking distant cortical regions while bypassing long chains of intermediate cortical processing (Sherman and Guillery, 2011; Cortes and Vreeswijk, 2015). This architecture has a natural computational analog in deep learning, where skip connections allow early feature representations to directly influence deeper processing stages, improving signal propagation and stabilizing hierarchical computations (Figure 1) (He et al., 2015). The analogy is especially compelling when the skip pathway is not merely additive, but *modulatory*: in biology, pulvinar-mediated influence is often described as gain control or context-dependent gating of cortical interactions; in machine learning, gated residual pathways similarly regulate the strength and content of long-range interactions.

Recent work on other HO nuclei further reinforces this view of the thalamus as a regulator of cortical dynamics. In particular, studies of the mediodorsal (MD) thalamus have shown that thalamocortical circuits can regulate cortical gain, stabilize task-relevant ensembles, and enable flexible switching between cortical representations during cognitive tasks (Schmitt et al., 2017; Rikhye et al., 2018; Mukherjee et al., 2021). Although these studies primarily concern prefrontal circuits, they support a broader view in which HO act as modulators of cortical dynamics rather than passive relays.

Despite extensive anatomical, physiological, and theoretical work describing pulvinar-mediated gating, precision control, and transthalamic routing (Shipp, 2003; Kanai et al., 2015; Sherman and Guillery, 2011; Cortes et al., 2026), the computational implications of these mechanisms remain largely unexplored in modern deep convolutional architectures. Most deep networks incorporate skip connections primarily to improve optimization and training stability (He et al., 2015; Veit et al., 2016), without explicit grounding in thalamocortical principles. Conversely, neurobiological accounts emphasize pulvinar-dependent routing, gain control, and interareal coordination, but the computational consequences of these mechanisms remain difficult to isolate experimentally (Zhou et al., 2016). A productive approach is therefore to formalize a minimal pulvinar-inspired pathway in a controlled deep learning framework and evaluate not only task performance but also mechanistic signatures such as representational stability and gain regulation (Cortes and Vreeswijk, 2012). This raises a fundamental question: does the pulvinar provide a biological blueprint for effective skip connections, and can integrating pulvinar-like pathways into artificial networks yield measurable computational advantages (Cortes et al., 2024, 2026; Sherman and Guillery, 2011; Saalmann and Kastner, 2011)?

Here we study a hierarchical vision model that includes a pulvinar-inspired pathway linking early and later stages of processing. This pathway allows information from early visual representations to influence later stages in a controlled manner, mimicking the modulatory role of the pulvinar in the brain. We evaluate this model in two complementary settings. First, in an image categorization task (CIFAR-10) (Krizhevsky, 2009), we compare a standard cortical network to a version that includes the pulvinar-like pathway. In this setting, we assess not only classification accuracy, but also how stable and well-scaled the internal representations remain when image contrast is varied. These measures allow us to determine whether pulvinar-like modulation primarily improves robustness and gain regulation, rather than simply boosting task performance. Second, we test the same models in a contrast-detection task designed to mimic near-threshold perceptual conditions (van Vugt et al., 2018), in which a faint target must be detected in a noisy background. By systematically varying target contrast, size, and background noise, this task probes how pulvinar-like modulation influences sensitivity when visual evidence is weak and uncertain. Together, these experiments provide a principled way to dissociate effects on representational stability and gain control from effects on perceptual sensitivity under noise.

## 2 Material and methods

### 2.1 Computational model

We modeled visual detection performance with a deep convolutional neural network (CNN) that approximates a cortical feedforward hierarchy, and an extended variant including a pulvino–cortical pathway. Let *x* ∈ ℜ^*H* × *W* × 3^ represents a real-valued RGB image, with spatial dimensions *H* × *W*, and three-color channels. For CNN input, grayscale stimuli were replicated across three channels to match the RGB input format. The baseline “cortical” network is a standard convolutional network with *L* layers. Denoting by *h*^(0)^ = *x* the input and by *h*^(*l*)^ the activity at layer *l*, the feedforward dynamics are:


$$\begin{array}{l} h^{l}\;=\;f_{l}\left(h^{\left(l-1\right)};\;\theta_{l}\right)=\;\sigma_{l}\left(W_{l}\;*h^{\left(l-1\right)}+\;b_{l}\;\right),\;l\;=\;1,...,L \end{array}$$ (1)

where ^*^ denotes convolution, σ_*l*_ is a pointwise non-linearity (ReLU), and θ_*l*_ = {*W*_*l*_, *b*_*l*_} are the trainable parameters. Here, *L*=3. The final read-out is computed from the last layer activity *h*^*L*^ as:


$$\begin{array}{lll} z\; & = & \;W_{out}\;\text{vec}\left(h^{\left(L\right)}\right)\;+\;b_{out}, \end{array}$$ (2)

$$\begin{array}{lll} y\; & = & \;\text{softmax}\left(z\right), \end{array}$$ (3)

Where, $\hat{y\;}\;\in {\left[0,1\right]}^{K}$ is the predicted probability over classes (e.g., target present vs. absent).

Training is performed by minimizing the cross-entropy loss with *L*_2_ regularization,


$$\begin{array}{l} L\left(\Theta \right)=\;-\frac{1}{N}\overset{N}{\underset{i=1}{\sum }}\overset{K}{\underset{k=1}{\sum }}y_{ik\;}\log{ŷ}_{ik}\;+\lambda ∥\Theta {{∥}^{2}}_{2} \end{array}$$ (4)

where Θ collects all trainable parameters in the network, *y*_*ik*_ is the one-hot encoded label for sample *i*, and λ is the weight-decay coefficient.

#### 2.1.1 Pulvino–cortical extension

To model the influence of the pulvinar on cortical processing, we introduced a parallel pulvino–cortical pathway that receives a copy of cortical activity from a mid-level “V1-like” layer and feeds back to a higher “decision-related” cortical layer. Concretely, we select two cortical layers **m** and **n (1 ≤ m < n** **≤** *L***)**. The pulvinar module receives as input a pooled representation of the cortical activity at layer **m**,


$$\begin{array}{l} c=\text{GAP}\left(h^{\left(m\right)}\right), \end{array}$$ (5)

Where, GAP (·) denotes global average pooling over spatial dimensions. The pulvinar state *p* is then computed as


$$\begin{array}{l} p\;=\;\phi_{p}\left(W_{pc}c+\;b_{p\;}\right), \end{array}$$ (6)

with ϕ_*p*_(·) a non-linear activation (ReLU) and parameters{*W*_*pc*_, *b*_*p*_}. This state is projected back to cortex through a pulvino–cortical projection,


$$\begin{array}{l} r\;=\;\phi_{c}\left(W_{cp}p+\;b_{r\;}\right), \end{array}$$ (7)

where ϕ_*r*_(·) is again a non-linearity and {*W*_*cp*_, *b*_*r*_} parameterize the projection, where *W*_*pc*_ implements a cortico-pulvinar transformation, capturing the convergence of early cortical activity onto the pulvinar, and *W*_*cp*_ models the reciprocal pulvino-cortical projection that modulates downstream cortical processing.

The resulting pulvino–cortical signal*r* has the same dimensionality as the target cortical layer activity *h*^(*n*)^ (up to reshaping), so that the combined activity at layer nnn is given by a convex combination


$$\begin{array}{l} {\tilde{h\;}}^{\left(n\right)}\;=\;\left(1-\alpha \right)h^{\left(n\right)}+\alpha r, \end{array}$$ (8)

Where, α ∈ [0, 1] is a gating parameter that controls the relative contribution of the pulvino–cortical input. For α = 0, the model reduces to the purely cortical baseline. For α > 0, the pulvinar pathway can amplify or reshape cortical representations at layer *n*.

In some simulations we additionally allowed for a learned gain on the pulvino–cortical feedback. In that case, the feedback is modulated by a gain vector*g* obtained from the pulvinar state,


$$\begin{array}{lll} g\; & = & \;\sigma_{g}\left(W_{g}p+\;b_{g\;}\right), \end{array}$$ (9)

$$\begin{array}{lll} \tilde{h} & = & h⊙s,\;\left(10\right) \end{array}$$ (10)

where σ_*g*_ is a sigmoid non-linearity and ⊙ denotes elementwise multiplication. This formulation interprets the pulvinar as dynamically modulating the precision of cortical signals by scaling the effective contribution of the feedback term.

All other layers *l* ≠ *n* are updated as in the cortical-only model. The pulvino–cortical model is trained end-to-end with the same loss function *L*(Θ); the additional parameters {*W*_*pc*_, *b*_*p*_, *W*_*cp*_, *b*_*r*_, *W*_*g*_, *b*_*g*_} are simply included in Θ. In the experiments reported here we compared a cortical baseline (α = 0)with pulvino–cortical models using different values of α and gain configurations.

##### 2.1.1.1 Squeeze-and-excitation control model (implementation baseline)

To test whether the observed effects are specific to the proposed pulvino-cortical mechanism or arise from generic channel-wise attention, we implemented a control model based on squeeze-and-excitation (SE) mechanisms.

In this model, modulation is applied locally within a single layer *h* as:


$$\begin{array}{l} s=\sigma \left(W_{2}\delta \left(W_{1}\text{GAP}\left(h\right)\right)\right), \end{array}$$ (11)

and


$$\begin{array}{l} \tilde{h}=h⊙s, \end{array}$$ (12)

where GAP is the global average pooling, δ is the ReLU non-linearity, σ is a sigmoid function producing channel-wise gains. SE is applied to the intermediate feature maps (block 2) and modulates activity multiplicatively before further processing.

##### 2.1.1.2 Projection-only skip control (non-modulatory control)

To isolate the computational contribution of long-range routing independently of modulation, we implemeted a control architecture consisting of a direct early-to-late projection without gating or adaptative fusion. This model preserves the same structural skip pathway used in the pulvino-cortical model but removes all modulatory components, including the gain gate *g* and the learned fusion parameter α.

So that, a projection is computed from an early cortical layer *h*^(*m*)^ to a deeper layer *h*^(*n*)^, using the same projection operator defined for the pulvino-cortical pathway:


$$\begin{array}{l} p=\Pi \left(h^{\left(m\right)}\right), \end{array}$$ (13)

where Π(·) is implemented as a lightweight transformation (convulation followed by spatial resampling) to match the dimensionality of *h*^(*n*)^, as described in Section 2.1.2.

In contrast to the pulvino-cortical model, the projected signal is combined with cortical activity throgh a fixed additive interaction:


$$\begin{array}{l} \tilde{h}=h^{\left(n\right)}+\beta p, \end{array}$$ (14)

where β is a constant scaling factor controlling the strength of the skip pathway. Importantly, β is not learned and remains fixed throughout training.

This formulation removes any stimulus-dependent modulation or adaptative weighting, ensuring that the skip pathway acts purely as feedforward projection. All other layers are uptdated as in the cortical baseline model. This control allows us to dissociate the effects of: i) long-range information routing, and ii) dynamic gain modulation introduced by the pulvino-coritcal mechanism.

By comparing this projection-only model with both the cortical baseline and the pulvino-cortical model, we can determine whether the observed effects arise from the presence of a skip pathway itself or from its modulatory properties.

#### 2.1.2 Architectural implementation (summary of Equations 5–10)

To implement the pulvino–cortical computations defined above, we added a long-range skip connection propagating activity from early to late stages of the CNN. Feature maps from the first convolutional block (64 channels at 16 × 16 resolution) were projected to match the input dimensionality of the third block (128 channels at 8 × 8 resolution) using a lightweight PulvinarProjector composed of a 1 × 1 convolution followed by bilinear interpolation.

**Figure 2:**
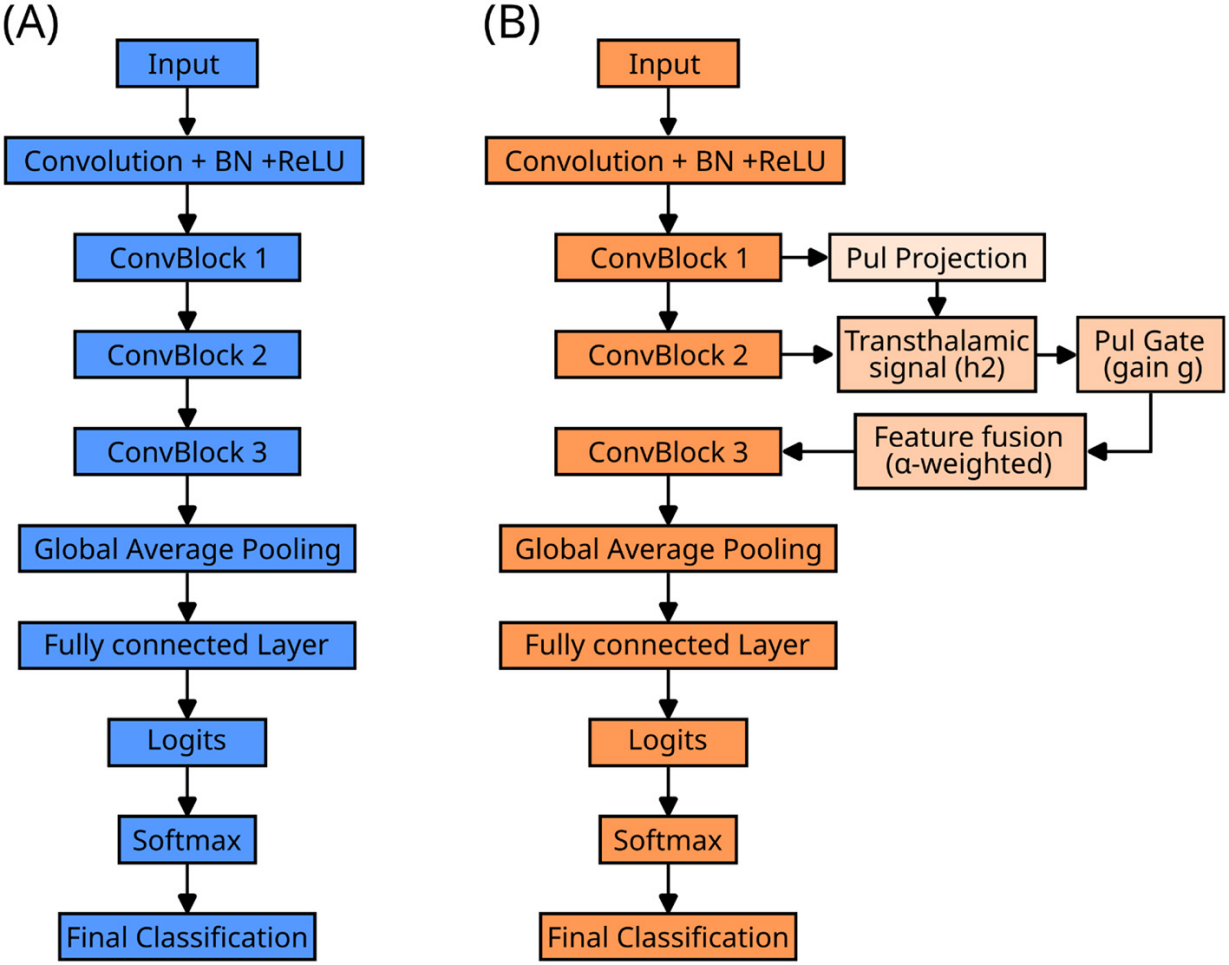
Network architecture. **(A)** Baseline cortical-only convolutional network. Visual input is processed through a hierarchy of convolutional blocks (ConvBlock 1–3), followed by global average pooling, a fully connected layer, and a softmax classifier producing the final decision. **(B)** Cortical–pulvinar network architecture. In addition to the feedforward cortical pathway, intermediate cortical representations are projected to a pulvinar module using a luminance-weighted 1 × 1 convolution, preserving spatial correspondence while reducing channel dimensionality. The pulvinar then generates a transthalamic signal (*h2*) that modulates cortical processing via a gain-control mechanism (*g*). Pulvinar-driven signals are reintegrated into the cortical stream through an α-weighted feature fusion stage prior to higher-level processing. The two architectures use identical classifier heads, allowing direct comparison of decision outcomes.

To capture the modulatory nature of pulvinar feedback, the projected signal passed through a PulvinarGate inspired by squeeze-and-excitation mechanisms, which computes a gating vector g that dynamically controls feedback strength.The gating vector g was computed using a squeeze-and-excitation–like mechanism: spatial global average pooling was applied to the projected feature maps to obtain a channel-wise descriptor, which was then passed through a two-layer fully connected network with a ReLU non-linearity, followed by a sigmoid activation to produce channel-wise gain values between 0 and 1. The gated pulvinar signal was combined with cortical activity additively, as formalized in Equations (9, 10). When gating was disabled, the gain was fixed to unity (g = 1), yielding a non-modulatory skip connection. Apart from this long-range pathway, all other architectural components were identical to those of the cortical baseline model (Figure 2).

**Task-specific model variants**. While Equations (5–10) define the full pulvino-cortical architecture; the effective instantiation of this mechanism differs across experimental paradigms due to task-dependent training constraints.

**1) CIFAR-10 categorization (Experiment 1):** the full model was used, including the pulvino–cortical projection, the gating mechanism (*g*), the fusion parameter (α), and the additional regularization terms λG, λα, and the target gain value G^*^, which constrained the statistics of the gating and fusion variables during training. These constraints were introduced to stabilize optimization and promote interpretable gain dynamics in a complex categorization setting.
**2) Near-threshold contrast detection (Experiment 2):** the same architectural core was retained, including gating mechanism (*g*), and fusion parameter (α). However, the regularization terms λG, λα, and the target gain G^*^ were removed to avoid imposing task-specific constraints on the gating dynamics, allowing the intrinsic behavior of the transthalamic pathway to emerge under controlled sensory uncertainty.

Thus, differences between Experiments 1 and 2 reflect differences in training regime rather than changes in architectural structure.

This distinction between architectural mechanism and training constraints is central to our approach. The pulvinar-inspired pathway defines a fixed computational structure (long-range routing with gain modulation), while the training regime determines how this mechanism is expressed in practice.

In this framework, the regularization terms used in Experiment 1 can be interpreted as shaping the operating regime of the network, rather than defining the mechanism itself. This separation allows us to distinguish between effects arising from representational constraints and those arising from the intrinsic properties of the architecture.

Accordingly, Experiment 1 includes two complementary analyses: a standard evaluation under the full training regime, and a control analysis in which models are compared within matched representational regimes.

This perspective motivates the control analyses introduced below, in which models are compared within matched representational regimes to isolate the contribution of the pulvino–cortical architecture independently of training-induced constraints.

#### 2.1.3 Training procedure

Both the cortical baseline and the pulvinar-augmented models were trained from scratch using identical optimization settings to ensure comparability. Training used the AdamW optimizer (learning rate ≈ 3 × 10^−4^, weight decay ≈ 1 × 10^−4^) with mini-batches of 128 images. Networks were trained for 20–50 epochs depending on configuration. A fixed random seed was used for all experiments. During training, loss and accuracy were recorded for both training and test sets, and TensorBoard was used for monitoring when available. All models were trained on GPU hardware when accessible; otherwise, computation defaulted to CPU. All simulations, training pipelines, and analyses were implemented in Python using PyTorch.

#### 2.1.4 Loss functions and training regimes

Across experiments, different training regimes were used to probe the interaction between architectural mechanisms and representational constraints.

The baseline cortical model was trained using standard cross-entropy loss:


$$\begin{array}{l} L_{CE}=CE\left(ŷ,y\right) \end{array}$$ (15)

In control analyses, we additionally introduced a contrast-based regularization term based on the S and HL metrics:


$$\begin{array}{l} L_{SH}=CE\left(ŷ,y\right)-\lambda SS-\lambda HHL \end{array}$$ (16)

For the pulvino–cortical model in Experiment 1, additional regularization terms were included to constrain the statistics of the gating and fusion mechanisms:


$$\begin{array}{l} L_{pulv,\;full}=CE\left(ŷ,y\right)-\lambda SS-\lambda HHL+\lambda GReg_{G}+\lambda \alpha Reg_{\alpha }, \end{array}$$ (17)

Where, Reg_G_ enforces a target gain level and Reg_α_ penalizes deviations from high fusion weighting.

In contrast, in Experiment 2 and in the architectural control analysis described above, these additional regularization terms (λG, λα, G^*^) were omitted to isolate the intrinsic contribution of the pulvino–cortical architecture.

### 2.2 Experiment 1: Image categorization (CIFAR-10)

All categorization experiments were conducted using the CIFAR-10 dataset (60,000 images; 10 classes). Images were normalized using standard channel statistics. Training images were augmented with horizontal flips and cropping; test images were normalized.

Both cortical baseline and pulvino–cortical models were trained from scratch using AdamW (learning rate ≈ 3 × 10^−4^, weight decay ≈ 1 × 10^−4^, batch size 128) for 20-30 epochs. Accuracy, loss, contrast sensitivity, and invariance metrics were computed on the test set.

Hyperparameters were optimized using classification accuracy on the validation set as the objective function. Importantly, the gain-linearity (HL) and directional invariance (S) metrics were not part of the optimization objective. Instead, they were computed *post hoc* on the trained networks corresponding to the accuracy-optimal parameter configuration.

#### 2.2.1 Contrast-perturbation set

During training of the pulvinar-augmented network, we introduced a contrast-based regularization computed from internal feature vectors. Given a mini-batch *x* and a set of contrast scaling factors *{*σ_1_*,…*,σ_*K*_*}* in our experiments {0.2,0.6,1.0}, we formed contrast-scaled inputs


$$\begin{array}{l} x^{\text{(k)}}=\sigma_{\text{k}}x,k=1,\ldots ,K \end{array}$$ (18)

Let *f*_θ_*(*·*)* denote the feature-extracting network parameterized by learnable weights θ. For each image *i* and contrast scaling σ*k*, we define


$$\begin{array}{l} u_{i}^{\left(k\right)}=f_{\theta }\left(x_{i}^{\left(k\right)}\right), \end{array}$$ (19)

Where $u_{i}^{\left(k\right)}\in {ℜ}^{256}$ denotes the pooled feature vector.

#### 2.2.2 S: direction invariance across contrast

We measured direction invariance as the mean cosine similarity between normalized feature vectors across all contrast pairs:


$$\begin{array}{l} S\;=\;\frac{1}{\left|P\right|}\;\underset{\left(a.b\right)\in P}{\sum }\left[\frac{1}{N}\;\overset{N}{\underset{i=1\;}{\sum }}\frac{<{{\bar{u}}_{i}}^{\left(a\right)},\;{{\bar{u}}_{i}}^{\left(b\right)}>}{{∥{\bar{u}}_{i}}^{\left(a\right)}∥\;∥{{\bar{u}}_{i}}^{\left(b\right)}∥}\right] \end{array}$$ (20)

$$\begin{array}{l} {{\bar{u}}_{i}}^{\left(k\right)}=\frac{{u^{\left(k\right)}}_{i}}{∥{u^{\left(k\right)}}_{i}\;∥} \end{array}$$ (21)

Where, *P* = {(*a, b*) | 1 ≤ *a* < *b* ≤ *K*} indexes all contrast pairs. Larger *S* indicates that representations preserve their direction in feature space under contrast changes.

#### 2.2.3 HL: signed gain linearity index

To quantify how feature magnitude changes with contrast, we computed the average feature norm per contrast level,


$$\begin{array}{l} F\left(\sigma_{k}\right)\;=\;\frac{1}{N}\;\overset{N}{\underset{i=1\;}{\sum }}∥{u_{i}}^{\left(k\right)}\;{∥}_{2}, \end{array}$$ (22)

then estimated a finite-difference slope between adjacent contrast levels and took its log:


$$\begin{array}{l} HL=\frac{1}{K-1}\;\overset{K-1}{\underset{k=1}{\sum }}\log\left(\max\left(\varepsilon ,\frac{F\left(\sigma_{k+1}\right)-F\left(\sigma_{k}\right)}{\sigma_{k+1}-\sigma_{k}}\right)\right), \end{array}$$ (23)

with ε a small constant to avoid log(0). Larger *HL* indicates a stronger (and more consistently positive) change in feature amplitude with contrast. Negative HL values indicate weak or sublinear scaling of feature magnitude with contrast, whereas positive HL values reflect robust gain modulation.

#### 2.2.4 Architectural control analysis within matched representational regimes

To dissociate the contribution of architectural mechanisms from training constraints, we performed an additional control analysis within the CIFAR-10 categorization task.

In this analysis, we compared multiple architectures under strictly matched optimization settings, including: (i) the cortical baseline model, (ii) a squeeze-and-excitation (SE) control model, (iii) a projection-only skip model, and (iv) the pulvino–cortical model. All models were trained using identical hyperparameters (learning rate = 3 × 10^−4^, weight decay = 1 × 10^−4^, label smoothing = 0.1, batch size = 128, and 100 training epochs), as well as identical data augmentation procedures. This procedure ensured that any observed differences arise from architectural mechanisms rather than optimization or regularization confounds.

To further isolate these effects, we evaluated performance within matched representational regimes defined by directional invariance (S) and gain linearity (HL). Specifically, we identified overlapping regions in the (S, HL) space across models and restricted comparisons to runs within this shared region [S ε (0.97, 0.99), HL ε (4.5, 5.5)].

Within this regime, accuracy differences reflect intrinsic architectural properties rather than differences in representational geometry.

### 2.3 Experiment 2: Near-threshold contrast detection paradigm

In this experiment, the pulvino-cortical model was evaluated without the λG, λα, and G regularization terms described in Table 1, allowing us to isolate the effect of the transthalamic architecture itself on near-threshold visual detection (while keeping the α fusion and gain gate parameters learned end-to-end). In contrast to Experiment 1, where these regularization terms were introduced to stabilize training and shape the statistics of the gating and fusion variables during a complex categorization task, the present experiment aims to directly assess how the architecture influences decision variables under controlled sensory uncertainty. Removing these constraints ensures that any observed effects on sensitivity (d′) or decision criterion arise from the intrinsic properties of the transthalamic pathway, rather than from task-specific training objectives.

**Table 1:** Global and pulvinar-specific hyperparameters and their usage across experiments.

Hyperparameter	Acts on	Diagram location	Role/effect	Used in experiment
Learning rate (lr)	All learnable weights	Global (training loop)	Controls update magnitude during optimization	Exp. 1 & 2
Weight decay (wd)	All learnable weights	Global (training loop)	Penalizes large weights (L2 regularization)	Exp. 1 & 2
Label smoothing	Output logits vs. targets	Output/Softmax	Reduces overconfidence	Exp. 1 & 2
α (learned)	Cortical-pulvinar feature fusion	Fusion block (layer n)	Learned, per-channel weighting of cortical vs pulvinar signals	Exp. 1 & 2
g (learned gate)	Dynamic feedback modulation	Pulvinar Gate	Learned, stimulus-dependent gain controlling pulvinar influence	Exp. 1 & 2
λα	α regularization	Fusion block	Regularizes α toward cortical-dominant regime	Exp. 1A only
G	Target average gating gain	Pulvinar Gate	Target mean gating level for g	Exp. 1A only
λG	Gain deviation penalty	Pulvinar Gate	Penalizes deviations of g from G	Exp. 1A only

The table summarizes training and model parameters, indicating their architectural location and functional role. Global hyperparameters are applied to all trainable weights. Pulvinar-related parameters controlled the strength and stability of the pulvino-cortical feedback pathway. λα, G, and λG were set to zero in Experiment 2.

#### 2.3.1 Stimulus generation

Synthetic grayscale stimuli consisted of a noisy background (mean luminance 0.5) and, on target-present trials, a faint circular target (van Vugt et al., 2018). Background noise was generated and filtered with a 1/*f* spectrum. Target circles of radius *R* were placed at random spatial locations and incremented by contrast *c* relative to background.

Contrast values ranged from 0.005 to 4%: 0.005%, 0.01%, 0.025%, 0.05%, 0.08%, 0.11%, 0.15%, 0.20%, 0.25%, 0.30%, 0.35%, 0.40%, 0.45%, 0.65%, 1.0%, 2.0%, 4.0%.

For each contrast level and parameter configuration, *N* = 1,000 stimuli were generated, with balanced target-present and target-absent trials.

#### 2.3.2 Experimental design

Background noise amplitude and target radius were systematically varied to probe different signal-to-noise regimes. For each configuration, the same cortical and pulvino–cortical models were trained on identical near-threshold stimuli to mimic a naïve observer and to ensure that behavioral and internal differences reflect architectural effects rather than training history. This design allows changes in false alarm rate and decision criterion to be interpreted as emergent properties of the transthalamic architecture, rather than as consequences of differential learning or stimulus familiarity.

Each network's binary output (target present / absent) was compared to ground truth to construct full confusion matrices, yielding the four canonical outcome categories: Hits (H), Misses / False Negatives (FN), False Alarms (FA), and True Negatives (TN). False positives (FP) were additionally analyzed as absolute counts to provide a more precise characterization of outcome redistribution beyond normalized false alarm rates.

Each network's binary output (target present/absent) was compared to ground truth to construct full confusion matrices, yielding Hits (H), Misses/False Negatives (FN), False Positives (FP), and True Negatives (TN). We report both FP counts and the corresponding false alarm rate (FA=FP/(FP+TN)), where FA provides a normalized SDT measure of decision bias and FP provides a count-level description of outcome redistribution across conditions. From these quantities, we computed: (i) accuracy; (ii) sensitivity (d′); (iii) decision criterion (c); and (iv) false alarm rate (FA). Psychometric detection curves were obtained by plotting hit rate (HR=H/(H+FN)) as a function of contrast for each (*R, BG*) configuration.

#### 2.3.3 Internal network responses and layer-wise analysis

To relate behavioral outcomes to internal network dynamics, we analyzed stimulus-evoked activity across corresponding layers of the two architectures. Three layers were selected as functional analogs of a hierarchical visual-decision pathway: an early layer (V1), an intermediate layer (V4), and a late decision-related layer (PFC). Decision-related modulation was quantified as:


$$\begin{array}{l} \Delta activation=activation_{\text{Hit}}-activation_{\text{Miss}} \end{array}$$ (24)

Positive Δactivation indicates stronger activity on Hits than Misses, whereas negative values indicate inverted or suppressive decision-related responses.

#### 2.3.4 Global analysis across stimulus space

All behavioral and internal measures were evaluated across the full grid of (*R, BG*). While overall accuracy was reported for completeness, primary analyses focused on FA and d′, which dissociate criterion shifts from genuine sensitivity changes within the SDT framework. Complete confusion-matrix statistics (H, FN, FP, TN, accuracy, d′, and criterion c) for all stimulus conditions are provided in the Supplementary material.

### 2.4 Summary of evaluated models and metrics

In Experiment 1, two model variants were compared: (i) a cortical baseline, and (ii) a jointly optimized cortical–pulvinar model. Performance was evaluated using classification accuracy, contrast sensitivity, and representational invariance. Table 1 summarizes the global and pulvinar-specific hyperparameters used during training, specifying the model components they affect, their location within the architecture, and their functional role. Global hyperparameters (e.g., learning rate, weight decay, and label smoothing) applied to all trainable weights, whereas pulvinar-specific parameters (λG, λα, and G) selectively regulated the strength, stability, and target level of modulatory feedback in the pulvinar skip-connection pathway. Hyperparameters in Experiment 1 were optimized using the Powell method (derivative-free optimization), implemented in Python.

In Experiment 2, the cortical baseline and the jointly optimized cortical–pulvinar model were evaluated in a near-threshold contrast detection paradigm. Background noise amplitude (BG) and target radius (R) were systematically varied to probe different signal-to-noise regimes. For each (BG, R) combination, the full range of contrast levels was generated, producing a dense set of near-threshold detection conditions. Both models were trained and evaluated under identical optimization settings, allowing a direct comparison of their sensitivity to weak visual signals.

### 2.5 Statistical analysis

Statistical analyses were performed using both parametric and non-parametric tests, selected according to data structure and sample size.

For Experiment 1A, differences between cortical-only and pulvino–cortical models were assessed using Welch's two-sample *t*-test, which does not assume equal variances. Effect sizes were quantified using Cohen's *d* to characterize the magnitude of model differences independently of sample size.

For Experiment 1B, which involved comparisons across multiple architectural conditions, we used the Kruskal–Wallis test to assess overall group differences without assuming normality or homogeneity of variance. When significant effects were detected, pairwise comparisons were performed using Dunn's *post hoc* test with multiple-comparison correction.

For analyses involving grid-based performance measures (*e.g.*, performance across combinations of stimulus size and background noise), comparisons between models were performed using the Wilcoxon signed-rank test. This non-parametric paired test was chosen because the number of grid points was limited, and normality assumptions could not be reliably satisfied.

To quantify condition-dependent enrichments, we additionally performed an abundance-style analysis in which “high” cases were defined using a one-sided z-threshold computed across the pooled stimulus grid (z > 1). This thresholding procedure was used to summarize the proportion of high-value cells by quadrant and was not treated as an inferential hypothesis test.

For grid-level comparisons across matched stimulus conditions (*BG, R*), we computed paired differences Δ=pulvinar–cortex (Pulvinar-cortical and cortical-only models) and tested whether the median shift differed from zero using the Wilcoxon signed-rank test. For metrics indexing decision bias (false alarms, false positives, and false negatives), we used one-sided Wilcoxon signed-rank tests reflecting the a priori hypothesis that pulvinar-like connectivity would increase liberal responding under near-threshold uncertainty. All other comparisons were assessed using two-sided tests.

## 3 Results

### 3.1.1 Experiment 1A: Image categorization (CIFAR-10). Model accuracy, invariance of tuning, contrast sensitivity and gain control, and hyperparameter optimization

**Figure 3:**
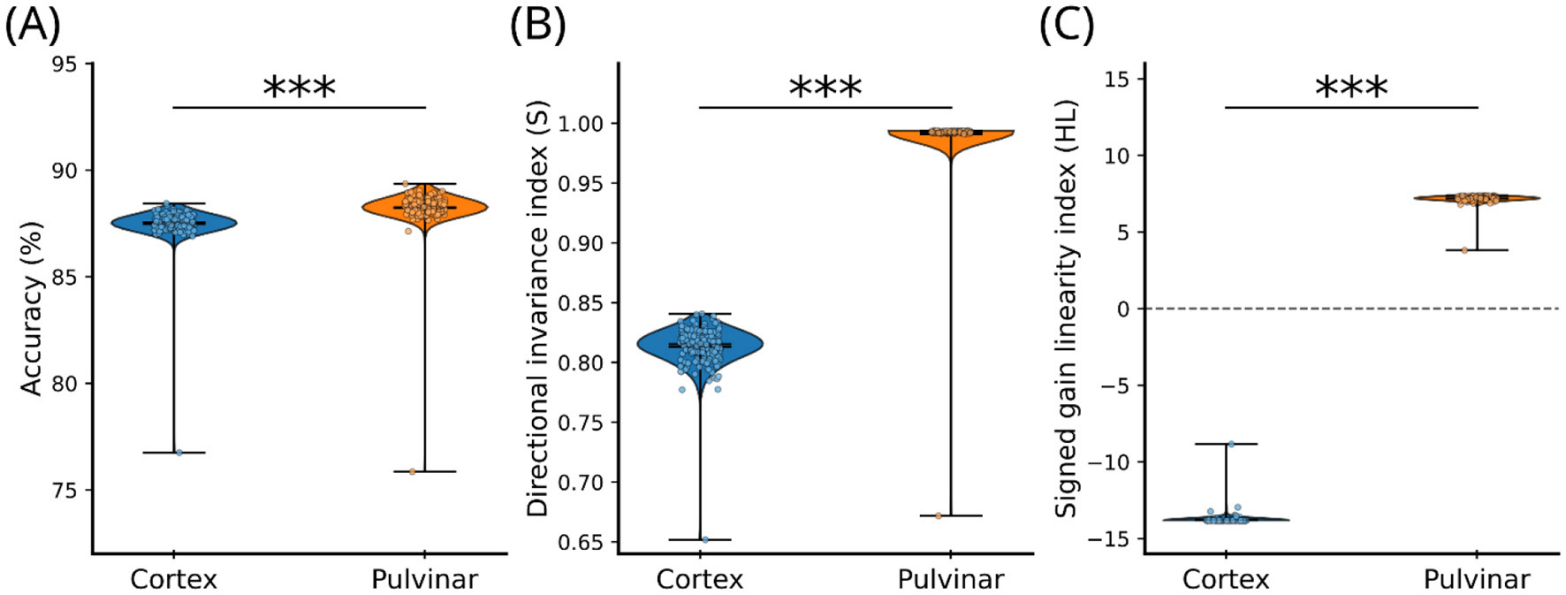
Comparison of cortical-only and pulvinar-augmented models on accuracy, invariance, and gain metrics. **(A)** Classification accuracy for cortical-only and pulvinar-augmented models. The pulvinar model shows a modest but significant improvement in accuracy. **(B)** Directional invariance index **(S)**, measuring the stability of internal feature representations across contrast perturbations. Pulvinar-like connectivity produces a large increase in representational invariance relative to the cortical baseline. **(C)** Signed gain linearity index (HL), quantifying contrast-dependent response scaling. Each point represents one trained model instance (*n*=100); violins indicate distributions across runs. Asterisks denote statistical significance (*** *p* < 0.001).

We compared a cortical baseline with a jointly optimized cortical–pulvinar model using independent hyperparameter searches (Figure 3; Tables 2, 3). In this experiment, the cortical baseline was trained using cross-entropy loss (Equation 15), whereas the pulvino–cortical model was trained using the full loss including S, HL, and gating/fusion regularization terms (Equation 17). Hyperparameters were optimized using classification accuracy on the validation set as the objective function. HL and S were subsequently evaluated on the model instance that achieved the highest validation accuracy, ensuring that these metrics reflect emergent properties of the architecture rather than optimization targets. The cortical baseline provided a reference level of categorization performance, whereas joint optimization of cortical and pulvinar parameters produced a small but reliable improvement in accuracy (Figure 3A, Table 4). Statistical analyses confirmed that this difference was significant but moderate in effect size (Cohen's d < 1), indicating that pulvinar-like modulation does not primarily act as a strong accuracy enhancer.

**Table 2:** Summary of analyses performed in Experiment 1.

Analysis/Experiment	Models	Loss	Hyperparameters matched?	Purpose
Exp. 1A	cortical vs. pulvinar	Original/full (Equation 17)	no	Functional regime
Exp. 1B	cortical, SE, skip, pulvinar	CE or CE–λS·S–λH·HL (Equations 15 and 16)	yes	Isolate architecture

Exp. 1A evaluates the cortical and pulvinar models under their original training configurations, reflecting the full functional regime shaped by both architecture and optimization constraints. Exp. 1B introduces a controlled comparison across architectures (cortical, SE, simple skip, and pulvinar) under matched hyperparameters and loss formulations (Equations 15–17), allowing isolation of architectural contributions independently of training-induced effects.

**Table 3:** Optimal hyperparameters and final performance scores for each model configuration.

Model	Optimized parameters	Accuracy (%)	HL (gain linearity)	S (direction invariance)
Cortical (baseline)	lr ≈ 3 × 10^−3^	87.47 ± 0.85	−13.76 ± 0.38	0.813 ± 0.017
	wd ≈4 × 10^−4^			
	ls ≈ 0.18			
Pulvinar + Cortical (joint)	lr ≈ 3.1 × 10^−3^	88.22 ± 0.96	7.18 ± 0.27	0.991 ± 0.023
	wd ≈ 4.1 × 10^−3^			
	ls ≈ 0.18			
	λG ≈ 0.01			
	λα ≈ 2 × 10^−4^			
	G^*^≈ 1.65			

The regularization parameters λG, λα, and the gain target G^*^ were used only in the CIFAR-10 categorization experiment (Section 4.1). The contrast detection experiment (Section 4.5) employed a simplified pulvinar pathway without these regularization terms.

**Table 4:** Between-model comparisons were performed using Welch's t-test for Accuracy, HL, and S.

Metric	Welch t	*p*-value	Cohen's d
Accuracy	7.91	3.21 × 10^−14^	0.82
HL (gain linearity)	608.47	< 1 × 10^−3^	63.75
S (invariance)	83.67	1.02 × 10^−229^	8.66

Effect sizes (Cohen's d) reveal that pulvinar-like modulation produces very large effects on gain control and representational stability, with only moderate effects on classification accuracy.

In contrast, the impact of pulvinar-inspired connectivity was much larger for gain-related and representational measures. As summarized in Table 2, the cortical–pulvinar model exhibited pronounced changes in contrast gain linearity (HL) and representational invariance (S), both associated with very large effect sizes (Cohen's d >> 1) (Table 4). This dissociation indicates that the principal computational contribution of the pulvinar pathway lies in restructuring how sensory signals are scaled and stabilized within the network, rather than in uniformly improving classification performance.

*Invariance of tuning*. Representational invariance was markedly enhanced in the cortical-pulvinar model relative to the cortical baseline (Figure 3B; Tables 3, 4), indicating more stable internal feature representations under contrast perturbations. This effect was robust and greatly exceeded the magnitude of the accuracy difference, supporting a role for pulvinar-like modulation in stabilizing cortical representations rather than directly optimizing categorization accuracy.

*Contrast sensitivity and gain control*. The cortical-pulvinar model also showed substantially improved contrast-dependent response scaling compared with the cortical baseline (Figure 3C; Tables 3, 4). The enhancement in gain linearity was highly reliable and associated with a very large effect size, consistent with a gain-control function of the pulvinar-inspired pathway that regulates how stimulus contrast is reflected in internal representations.

Together, these results reveal a clear dissociation between accuracy and gain-related effects. While pulvinar-like modulation yields only modest improvements in classification accuracy, it produces strong and systematic changes in contrast gain and representational stability. This pattern indicates that the primary computational impact of the pulvinar pathway lies in regulating gain and stabilizing internal representations, rather than acting as a direct accuracy booster.

### 3.1.2 Experiment 1B: Controlled architectural comparison and representational regime

To determine whether the computational effects attributed to the pulvinar pathway arise from its specific architecture rather than optimization constraints or generic mechanisms, we compared four model configurations: a cortical-only baseline, a pulvinar-cortical model, a squeeze-and-excitation (SE) model, and a simple skip connection without modulation. The SE model was included to test whether the observed effects could be explained by a standard attention-like mechanism, while the simple skip connection tested whether they could arise from the addition of a parallel pathway alone. All models were trained using identical optimization parameters (learning rate, weight decay, and label smoothing), ensuring a fair comparison of architectural contributions. Unlike Experiment 1A, pulvinar-specific regularization terms (Equation 17) were removed. Models were trained either with standard cross-entropy loss (Equation 15) or with additional S and HL regularization (Equation 16), allowing us to compare architectures both in unconstrained and regime-controlled settings.

**Figure 4:**
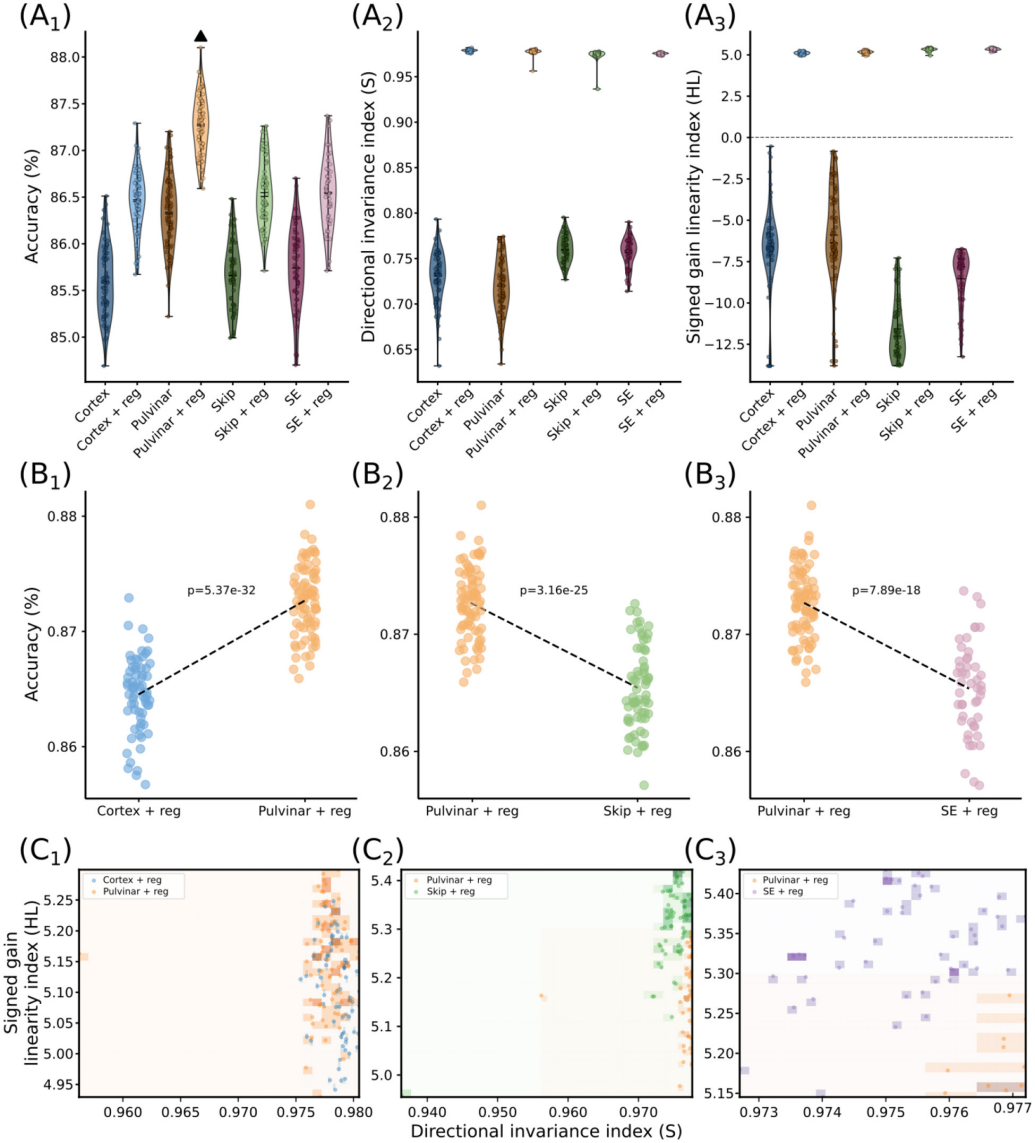
Controlled comparison of model architectures and representational regimes. **(A)** Distribution of test accuracy **(A1)**, directional invariance **(S; A**_**2**_**)**, and gain linearity **(HL; A3)** across models. Regularization drives all models toward similar S and HL values, indicating control by the loss, yet only the pulvino–cortical model achieves the highest accuracy (Black triangle) (*p*-values of Dunn *post-hoc* tests in Supplementary Tables 3–5). **(B)** Accuracy comparisons restricted to matched S–HL regions show that the pulvinar model consistently outperforms the cortical baseline **(B1)**, simple skip **(B**_**2**_**)**, and SE **(B3)** models (*p*-values indicated), demonstrating that performance differences cannot be explained by representational regime alone. **(C1–C3)** Overlap of model distributions in S–HL space after restriction. Points represent individual runs and shaded regions with their density. The baseline and skip models overlap with the pulvinar model, whereas the SE model occupies a distinct region, indicating that the pulvinar advantage arises from how the architecture operates within a shared regime.

Across configurations, the regularization terms consistently shape the representational metrics S and HL (Figure 4A). Models trained with the extended loss converge to similar ranges of S and HL, indicating that these quantities are primarily controlled by the optimization constraints rather than the architecture itself. However, despite this convergence in representational space, only the pulvino–cortical model translates this regime into a systematic improvement in accuracy. The baseline, simple skip, and SE models reach comparable S–HL values but do not achieve the same performance. This dissociation indicates that regularization alone is not sufficient: the advantage arises from how the pulvinar architecture operates within this controlled regime.

To directly test whether performance differences could be explained by the representational regime, we first restricted the analysis to runs falling within specific S and HL ranges, thereby defining a controlled region of the S–HL space (Figure 4B). This ensures that all models are evaluated under comparable representational conditions. Within this restricted subset, the pulvinar model with regularization consistently outperforms the baseline, simple skip, and SE models that also included regularizations.

We next examined the S–HL space defined by this restriction to verify whether models operate within a shared regime. As shown in Figure 4C, this procedure defines regions of overlap between models. In particular, the cortical-alone and pulvinar models, as well as the simple skip and pulvinar models, exhibit clear overlap within the restricted S–HL space, confirming that these comparisons are performed under matched conditions. In contrast, the pulvinar and SE models occupy largely distinct regions, indicating that they operate under different effective regimes.

This dissociation shows that performance differences cannot be explained by representational geometry alone but instead reflect how each architecture operates within (or across) these regimes. Together, these results indicate that the pulvinar-inspired pathway implements a specific computational mechanism that cannot be reduced to optimization, generic attention, or the addition of a parallel pathway, but instead reflects a distinct form of long-range routing and gain modulation.

### 3.2 Contrast detection near-threshold paradigm

Although CIFAR-10 categorization revealed only modest accuracy differences between cortical and cortical–pulvinar models, pulvinar-like modulation produced strong effects on gain control and representational stability (Figure 3, Table 4). To probe the functional consequences of these effects under sensory uncertainty, we evaluated the same pulvino-cortical architecture in a near-threshold contrast-detection paradigm. Importantly, this experiment follows the controlled regime introduced in Experiment 1B: models were trained under identical optimization conditions and without pulvinar-specific regularization terms (λG, λα, G^*^), isolating the intrinsic contribution of the transthalamic pathway (Table 1). This ensures that any observed effects cannot be attributed to training constraints but reflect architectural properties. The task required detecting a faint circular target embedded in a noisy background, while target contrast, target size, and background noise amplitude were systematically varied. This paradigm enabled us to assess how pulvinar-inspired modulation influences sensitivity to weak visual signals and the balance between signal and noise—effects that cannot be inferred from standard categorization accuracy alone.

#### 3.2.1 Response to contrast and perceptual detection

Perceptual detection performance was evaluated in two network architectures: a cortical model consisting of a convolutional neural network (CNN) alone, and a pulvinar-cortical model incorporating skip connections to emulate thalamocortical interactions. Model responses were analyzed under four representative stimulus conditions defined by stimulus size (radius, *R*) and background noise level (*BG*): low noise (*BG* = 0.25) with small (*R* = 3) or large (*R* = 13) stimuli, and high noise (*BG* = 2.5) with small or large stimuli. For each condition, psychometric detection curves, distributions of sensitivity (*d*′), and full confusion matrices are shown to preserve the structure of perceptual decision outcomes.

**Figure 5:**
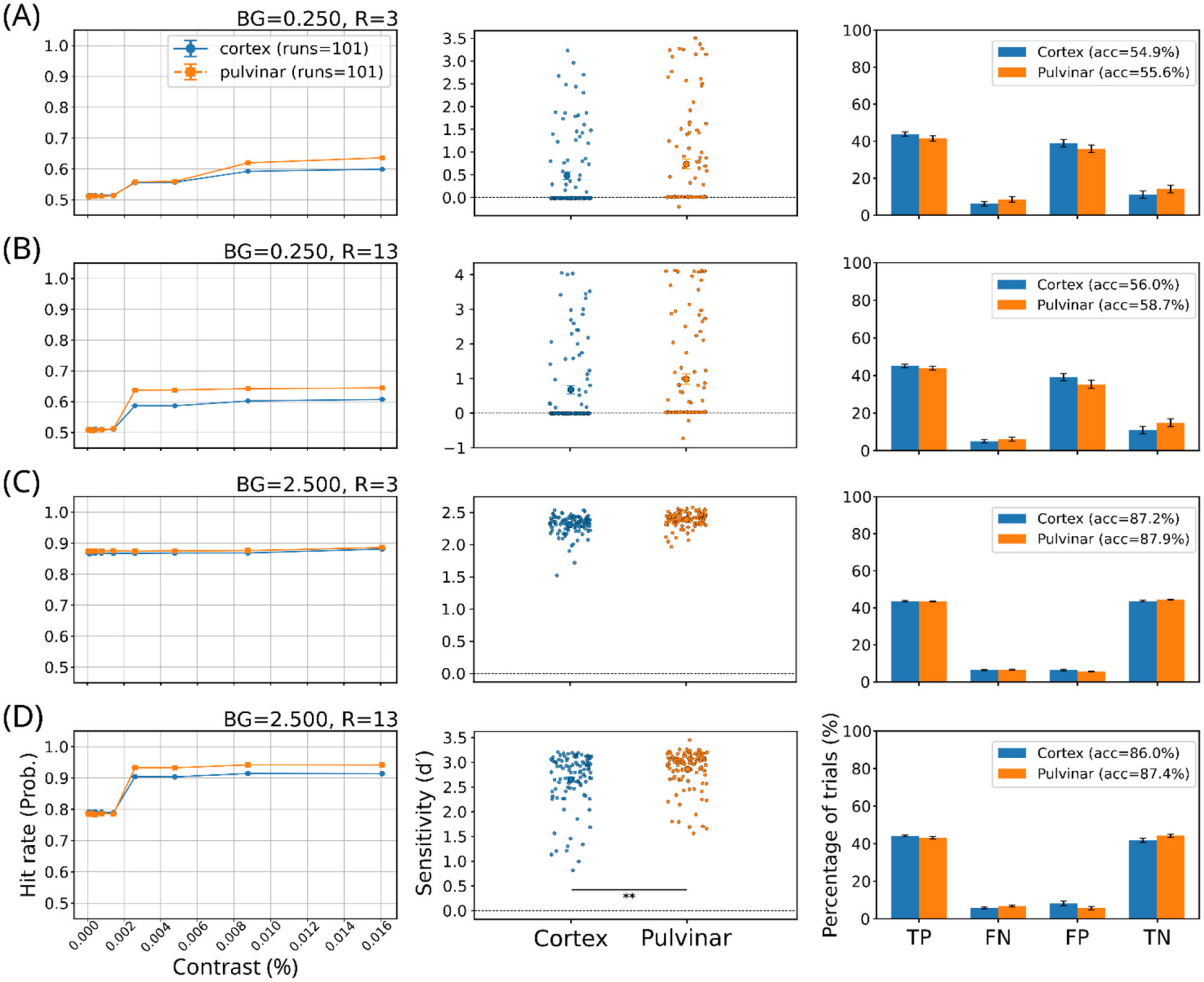
Near-threshold contrast detection performance across stimulus conditions. Rows correspond to four representative stimulus configurations defined by background noise amplitude (BG) and target radius (R): **(A)** BG = 0.25, R = 3; **(B)** BG = 0.25, R = 13; **(C)** BG = 2.5, R = 3; and **(D)** BG = 2.5, R = 13. Left panels show psychometric curves (hit rate as a function of contrast) for cortical-only and pulvinar-augmented models (mean ± SEM across runs). Middle panels show distributions of sensitivity (d′) across model instances for the same conditions, with each point representing one run. Right panels show confusion-matrix outcome proportions (true positives, false negatives, false positives, and true negatives), with overall accuracy indicated in the legend. Asterisks denote statistically significant differences between architectures (** *p* < 0.01).

Under low background noise (*BG* = 0.25), both small and large stimuli yielded near-chance performance (Figures 5A, 5B). Psychometric curves were shallow and largely flat across contrast levels for both architectures, with overall accuracies close to 55-59%. Although the pulvinar-cortical model showed a slight upward shift in the psychometric curves relative to the cortical model, *d*′ values were highly variable and largely overlapping between networks. Confusion matrices revealed small redistributions of outcomes, typically a mild increase in hits accompanied by a corresponding increase in false alarms, without a consistent gain in discriminability.

In contrast, under high background noise (*BG* = 2.5), detection performance improved markedly for both architectures (Figures 5C, D). For the small-radius stimulus (*R* = 3), psychometric curves were high and nearly saturated, and both models achieved similar accuracies (~87-88%). Sensitivity (*d*′) distributions largely overlapped, and confusion matrices showed balanced increases in true positives and true negatives, indicating robust detection without a clear architectural advantage.

A distinct pattern emerged in the high-noise, large-radius condition (*BG* = 2.5, *R* = 13). Here, the pulvinar–cortical model exhibited a significant increase in sensitivity (*d*′) relative to the cortical-only model (Figure 5D, middle panel), despite only a modest difference in overall accuracy (87.4% vs. 86.0%). Importantly, confusion matrices revealed that this increase in *d*′ was accompanied by a systematic redistribution of decision outcomes: the pulvinar-cortical network showed both increased hit rates and reduced false alarms, together with fewer false negatives. This pattern indicates an improvement in discriminability that cannot be attributed solely to a liberal shift in decision criterion.

Taken together, these results show that pulvinar-like interactions do not uniformly enhance perceptual sensitivity across stimulus regimes. In most conditions, the pulvinar–cortical model primarily induces noise-dependent changes in the distribution of detection outcomes, consistent with a modulation of decision criterion. However, under conditions of high sensory uncertainty combined with strong spatial integration demands (large *R*, high *BG*), pulvinar-like connectivity can also support a genuine increase in perceptual sensitivity, as reflected by elevated *d*′. This suggests that the pulvinar contribution is conditional and emerges most clearly when global stimulus integration is required to stabilize perceptual decisions under noise.

#### 3.2.2 Response of networks as a function of layers

We examined stimulus-evoked activity across network layers under the same stimulus size (*R*) and background noise (*BG*) conditions used for behavioral analyses, to identify the internal activity patterns associated with detection outcomes. Activations were compared between the cortical-only and pulvinar–cortical models across three corresponding layers (V1, V4, and PFC), used here as analogs of a hierarchical processing stream.

Layer-specific activity was analyzed as a function of behavioral outcome by computing the difference in unit activation between Hit and Miss trials (Δactivation = Hit – Miss). Positive Δactivation indicates stronger activity on Hit than Miss trials, whereas negative Δactivation indicates the opposite.

**Figure 6:**
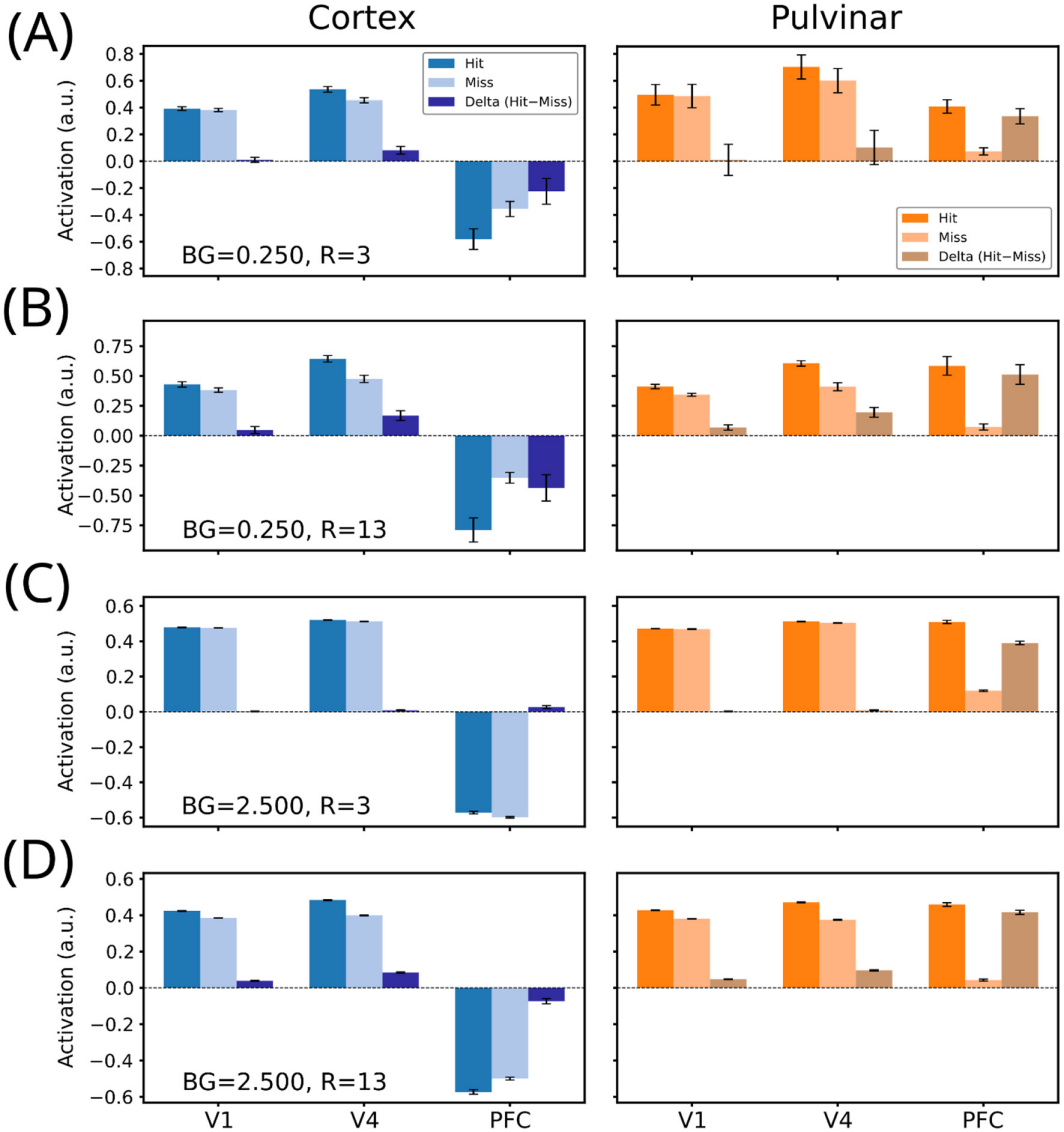
Layer-specific decision-related activity in cortical and pulvinar-augmented networks across stimulus conditions. Mean activations in V1, V4, and PFC-like layers are shown for Hit and Miss trials, together with their difference (Δactivation = Hit – Miss), under four combinations of background noise (BG) and target radius **(R)**: **(A)** BG = 0.25, R = 3; **(B)** BG = 0.25, R = 13; **(C)** BG = 2.5, R = 3; and **(D)** BG = 2.5, R = 13. Early (V1) and intermediate (V4) layers show relatively small and comparable Hit–Miss separation across architectures. In contrast, the late PFC-like layer exhibits an architecture-dependent effect: pulvinar-augmented networks display positive Hit-selective activity, whereas cortical-only networks show weak or inverted decision-related responses. These results indicate that pulvinar-like modulation primarily reshapes late-stage decision representations rather than early sensory processing.

*Low background noise (BG* = *0.25)*. For both stimulus sizes (*R* = 3 and *R* = 13), V1 and V4 showed positive Hit-Miss separation in both models, with Δactivation being small in V1 and larger in V4 (Figures 6A, B). The major divergence between the two architectures emerged at the PFC level. In the cortical-only model, PFC activations were strongly negative, and importantly, more negative for Hits than for Misses, producing a large negative Δactivation (Figures 6A, B, cortex PFC). In contrast, in the pulvinar-cortical model, PFC activity was positive for Hits and near-baseline for Misses, yielding a large positive Δactivation (Figures 6A, B, pulvinar PFC). Thus, under low-noise conditions, the pulvinar-like skip connections induce a qualitative inversion of PFC decision-related activity, switching from a strong negative Hit-associated signal in the cortical model to a strong positive Hit-associated signal in the pulvinar-cortical model.

*High background noise (BG* = *2.5)*. Under high noise, V1 and V4 Hit and Miss activations were nearly overlapping in both architectures, resulting in Δactivation values close to zero in these layers (Figures 6C, D). Again, the main model-dependent effect was observed in PFC. In the cortical-only model, PFC activity remained negative for both Hits and Misses, and Δactivation was small (near zero, slightly positive for *R* = 3 and slightly negative for *R* = 13; Figures 6C, D, cortex PFC). In contrast, the pulvinar–cortical model showed positive PFC activation for Hits and a much lower (near-baseline) activation for Misses, producing a substantially larger positive Δactivation in both stimulus sizes (Figures 6C, D, pulvinar PFC).

In summary, across conditions, V1 and V4 showed modest or minimal Hit–Miss separation and were similar across architectures, whereas PFC exhibited the strongest divergence. The pulvinar-cortical model produced positive, Hit-selective PFC activity, while the cortical-only model showed predominantly negative or weakly differentiated responses. Together, these results suggest that pulvinar-like input primarily reshapes late-stage decision-related representations, biasing PFC toward stable, decision-consistent activity states and modulating the decision criterion rather than sensory sensitivity.

#### 3.2.3 Global response of networks to contrast and perceptual detection

To characterize how the observed behavioral differences depend on stimulus conditions, we examined network responses across the full space of stimulus size (R) and background noise (BG). For each parameter combination, we computed matrices summarizing confusion-matrix variables as well as signal detection measures. Although overall accuracy was largely comparable between cortical-only and pulvinar-cortical networks across conditions, accuracy alone is insensitive to redistributions of decision outcomes that reflect changes in decision criterion. We therefore focused our analysis on false alarm rate (FA) and sensitivity (d′), which respectively index decision bias and signal–noise separability within the signal detection framework. To streamline the presentation and highlight the most informative effects, we report here only the matrices for FA and d′. Complete results for all confusion-matrix components, including accuracy, true positives, false negatives, and true negatives, are provided in the Supplementary material.

To facilitate interpretation, stimulus conditions were grouped into four representative quadrants defined by BG and R: Q1, low background noise with small stimuli; Q2, high background noise with small stimuli; Q3, low background noise with large stimuli; and Q4, high background noise with large stimuli. Throughout this section, Δ denotes the paired difference between architectures (pulvinar–cortical minus cortical-only), computed for each stimulus condition before statistical testing.

**Figure 7:**
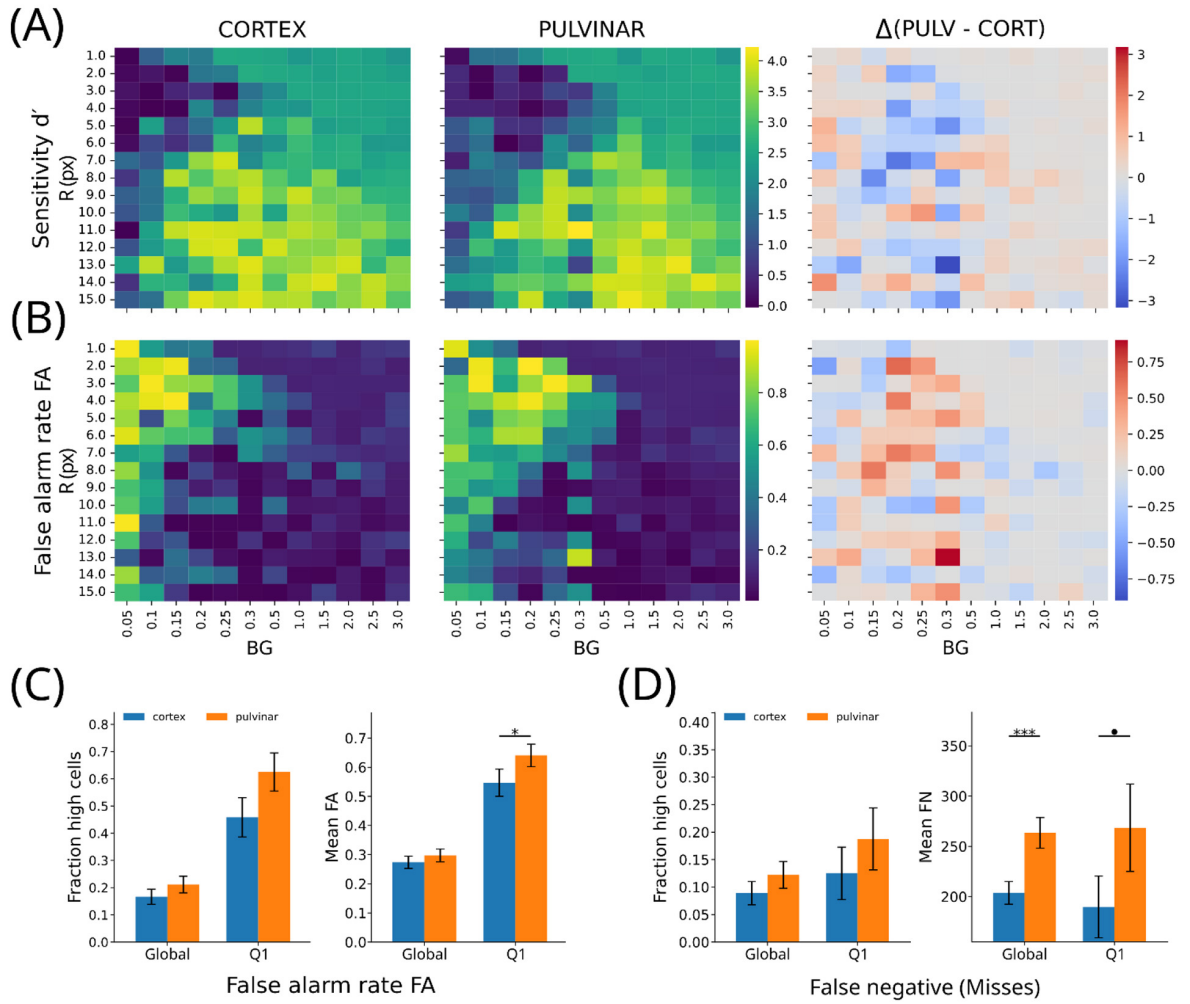
Global stimulus-dependent effects of pulvinar-like modulation on sensitivity and decision bias. **(A)** Heatmaps of sensitivity (d′) across the stimulus grid defined by target radius **(R)** and background noise amplitude (BG) for cortical-only **(left)**, pulvinar–cortical **(middle)**, and their difference (Δ = pulvinar – cortical; right). Sensitivity varies primarily with stimulus regime, with limited global advantage for pulvinar-like connectivity. **(B)** Corresponding heatmaps of false alarm rate (FA), showing a selective increase in FA in the pulvinar–cortical model under low-signal conditions. **(C)** Summary statistics for FA: fraction of high-FA stimulus cells (z > 1) and mean FA, computed globally and within the near-threshold quadrant (Q1: low BG, small R). Pulvinar-like modulation significantly increases false alarms in Q1, consistent with a liberal criterion shift. **(D)** Summary statistics for false negatives (FN), showing increased FN counts in the pulvinar–cortical model, particularly in Q1. (*p* < 0.1; *p < 0.05; ***p < 0.001).

Across stimulus conditions, pulvinar-augmented networks did not exhibit a uniform improvement in perceptual sensitivity or overall accuracy, as indexed by d′ (Figure 7A). Instead, their most prominent effect was a systematic increase in false alarm rate under low-signal conditions (Figure 7B). This effect was most pronounced in Q1, corresponding to small stimuli under low background noise, a near-threshold regime in which object detection is intrinsically difficult, and sensory evidence is weak.

To quantitatively compare networks across conditions, we identified stimulus configurations in which FA values exceeded the global mean by more than one standard deviation (z-score > 1), computed across the full stimulus grid. Under this criterion, the pulvinar-cortical model exhibited a larger proportion of high-FA cases than the cortical-only network at the global level (cortical-only: 16.7%; pulvinar-cortical: 21.1%; Figure 7C). This difference was substantially amplified in Q1, where high-FA cases were markedly more frequent in the pulvinar-cortical network (cortical-only: 45.8%; pulvinar-cortical: 62.5%; Figure 7C).

Consistent with this pattern, pooling responses across all stimulus conditions revealed a modest but significant redistribution of decision outcomes in the pulvinar–cortical model. This global effect was characterized by a reduction in hit rate and perceptual sensitivity together with an increase in false negatives (TP: Δ = −59.6, *p* < 0.001; H/d′: Δ = −0.024, *p* < 0.001; FN: Δ = + 59.6, *p* < 0.001; Figure 7D, Supplementary Table 1), while false alarm and false positive rates did not increase at the global level. Thus, when averaged across stimulus conditions, pulvinar-like connectivity shifts the overall balance of responses without inducing indiscriminate or stimulus-independent detections.

Crucially, this global averaging masked strong condition-dependent effects. In Q1, the pulvinar–cortical network exhibited a selective increase in false alarms and false positives (FA: Δ = + 0.094, *p* = 0.026; FP: Δ = + 235, *p* = 0.026; Figure 7C), without a corresponding increase in hit rate or sensitivity. This pattern is consistent with a localized liberal shift in decision criterion under weak sensory evidence, rather than an improvement in perceptual discriminability. Importantly, this increase in false alarms was accompanied by an increase in false negatives (cortical-only: 8.9% vs. pulvinar–cortical: 12.2%; Δ = +78.7, *p* = 0.05; Figure 7D, Supplementary Table 1), while true positive counts did not increase disproportionately and were in some conditions even higher in the cortical-only model.

By contrast, in Q3 (large stimuli under low background noise), the dominant effect was a marked increase in false negatives together with reduced hit rate and sensitivity (FN: Δ = +169, *p* < 0.001; Supplementary Table 1), indicating impaired discriminability when large-scale spatial integration was required. Differences between architectures were minimal under higher background noise regimes (Q2 and Q4), with Q4 showing an increase in true negatives (Δ = +73, *p* < 0.001; Supplementary Table 1), consistent with more conservative responding under high noise.

Taken together, these results indicate that pulvinar-like connectivity does not induce stimulus-independent false percepts. Rather, it modulates the balance between signal and noise by shifting the decision criterion in a stimulus-dependent manner, producing localized increases in false alarms under near-threshold conditions while reshaping the hit–miss balance in regimes that place high demands on perceptual integration.

#### 3.2.4 Global response of networks as a function of layers

We examined how activity propagates across network layers by analyzing layer-specific responses to identical visual inputs while varying target R and BG. As in Section 3.2.2, for each layer, stimulus-evoked responses were quantified using Δactivation in early (V1), intermediate (V4), and late (PFC) layers for both cortical-only and cortical–pulvinar architectures.

**Figure 8:**
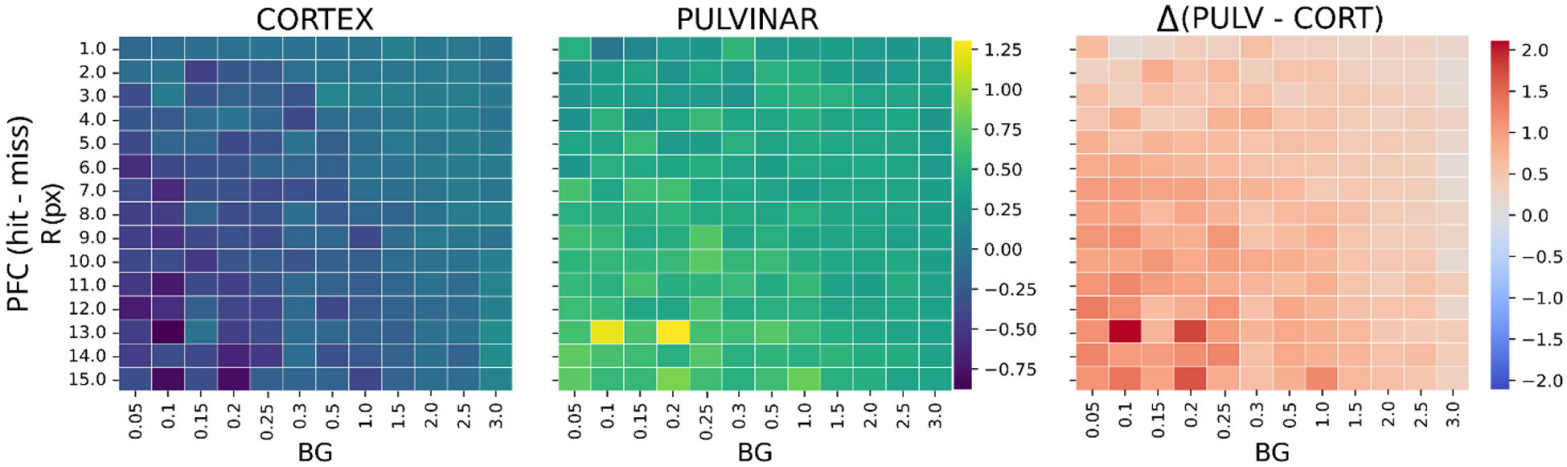
Global activation of PFC for the two networks as a function of target radius **(R)** and background noise (BG). Each unit represents the difference between Hit and Miss responses (Δactivation). Left: cortical-only model. Middle: pulvinar–cortical model. Right: paired difference (pulvinar – cortex). Pulvinar-like modulation produces positive Hit activity across conditions.

Across conditions, Δactivation in V1 and V4 was similar between architectures, with only modest modulation by stimulus size or noise and no consistent sign differences (Supplementary Figure 1). In contrast, strong differences emerged in PFC (Figure 8). In the cortical-only network, PFC activity was predominantly negative across conditions, yielding weak or inverted Hit–Miss separation that was largely invariant to R and BG. By comparison, the cortical–pulvinar network consistently produced positive PFC responses, resulting in positive Δactivation values that were strongest for larger targets and low background noise, and gradually decreased with increasing stimulus uncertainty.

Together, these results indicate that pulvinar-like input biases PFC activity toward decision-consistent states, consistent with a modulation of decision criteria rather than early sensory encoding.

## 4 Discussion

The present study examined the computational consequences of embedding a minimal, biologically inspired pulvinar-like pathway into a hierarchical convolutional vision model. We introduced a long-range transthalamic skip connection that projects early visual feature maps to later, decision-related layers through a lightweight projection module whose influence is dynamically regulated by a gain-controlled gating mechanism. This design dissociates long-range information routing from local feedforward processing, capturing a core functional motif attributed to the pulvinar in thalamocortical circuits: modulation of cortical interactions without direct encoding of sensory content (Sherman and Guillery, 2011; Saalmann et al., 2012; Cortes et al., 2021).

Using this architecture, we evaluated two complementary computational regimes. In a standard object categorization task (CIFAR-10), we assessed how pulvinar-like modulation reshapes internal representations under contrast perturbations beyond its effect on classification accuracy. In a near-threshold contrast-detection paradigm under controlled noise, we examined how the same transthalamic pathway influences perceptual decisions when sensory evidence is weak and uncertain. Across both tasks, a consistent pattern emerged: pulvinar-inspired connectivity exerted its strongest effects on gain regulation and decision criterion rather than on raw sensitivity or accuracy. These effects arose downstream of sensory encoding, at the level of evidence weighting and decision formation (Summerfield and Tsetsos, 2015; Heekeren et al., 2008).

Although the pulvinar-augmented network produced only modest improvements in categorization performance, it induced large and systematic changes in contrast-dependent response scaling and representational stability. In the near-threshold detection task, pulvinar-like modulation primarily redistributed decision outcomes in a stimulus-dependent manner, consistent with shifts in decision criterion, while genuine increases in sensitivity (d′) emerged only under restricted conditions combining high uncertainty and strong spatial integration demands. Together, these findings support a view of the pulvinar as a regulator of how sensory evidence is weighted and converted into decisions, rather than as a direct enhancer of sensory encoding or perceptual sensitivity (Feldman and Friston, 2010; Heekeren et al., 2008).

While the proposed pulvinar-like pathway shares superficial similarities with attention mechanisms such as squeeze-and-excitation (Hu et al., 2018), its computational role is fundamentally distinct. Standard attention modules typically reweight feature channels within a given layer to enhance task-relevant representations (Vaswani et al., 2017). In contrast, the present architecture modulates the effective strength of a fixed, long-range communication pathway between early and late processing stages, based on a compressed summary of early activity. Rather than performing feature selection, this mechanism regulates gain, stability, and the influence of early sensory representations on downstream decision processes. This distinction highlights the role of the pulvinar-inspired pathway as a biologically grounded routing-and-gain control mechanism, rather than a conventional attention module.

In the following sections, we first relate the network architecture to known anatomical and functional properties of cortico–pulvinar circuits and then examine the task-specific computational effects that emerge from this design.

### 4.1 Anatomical and functional considerations in network architecture

The pulvinar-inspired architecture incorporates a long-range skip connection in which higher cortical layers provide a dynamic gating signal that modulates pulvinar feedback to cortex. Although uncommon in standard artificial networks, this bidirectional and asymmetric interaction is well grounded in thalamocortical anatomy (de Souza et al., 2021; Abbas Farishta and Casanova, 2020; Cortes et al., 2021; Miller-Hansen and Sherman, 2022). Cortico–pulvinar projections vary systematically along the cortical hierarchy: lower visual areas predominantly provide driver-like inputs, whereas higher-order cortical areas send more modulatory projections. This organization supports a functional distinction in which the pulvinar integrates feedforward sensory signals while receiving contextual and regulatory input from higher cortical regions.

The present model captures this principle at an abstract level by allowing higher-layer activity to regulate transthalamic feedback strength, without explicitly modeling the full diversity of driver and modulator synapses. While simplified, this framework isolates the computational consequences of hierarchical cortico–pulvinar interactions. Future extensions could incorporate multiple projection classes and layer-specific dynamics to more closely reflect known anatomical and physiological properties of the pulvinar (Saalmann et al., 2012; de Souza et al., 2021).

### 4.2 Effects of pulvinar-like modulation on visual discrimination

Across evaluation metrics, the jointly optimized cortical–pulvinar model exhibited a more balanced representational profile than the cortical-only baseline. While accuracy gains were modest and stimulus-size dependent, the strongest effects emerged at the level of internal representational geometry.

Pulvinar-inspired connectivity increased contrast invariance, indicating that feature-vector direction was preserved across changes in stimulus intensity (Figure 3B). This reflects stabilization of representational geometry, whereby contrast modulates activation magnitude without disrupting the structure used for downstream readout (DiCarlo et al., 2012). Complementing this effect, pulvinar-like modulation induced a qualitative shift in contrast-dependent gain, captured by the contrast–linearity metric (HL) (Figure 3C). Whereas, the cortical-only network exhibited a compressive regime that limited dynamic range, the pulvinar-augmented model restored a more linear relationship between contrast and feature norm. This controlled rescaling parallels physiological evidence that pulvinar input regulates contrast gain in visual cortex (de Souza et al., 2020).

Together, these results indicate that pulvinar-like modulation reshapes cortical representations by stabilizing geometry while regulating gain. Rather than enhancing discrimination through sharper selectivity, the pulvinar-inspired pathway preserves scalable and interpretable representations, consistent with theoretical accounts of transthalamic gain control and controlled signal propagation in hierarchical systems (Cortes and Vreeswijk, 2012, 2015).

### 4.3 Control analysis and regime interpretation

To ensure that these effects cannot be attributed to generic architectural features or optimization constraints, we compared the pulvinar model to control architectures including a squeeze-and-excitation (SE) module and a simple skip connection (Figure 4). Although all models were trained under identical conditions and converged to similar S and HL values, only the pulvino–cortical architecture translated this regime into improved performance.

Restricting the analysis to matched regions of the S–HL space confirmed that this advantage persists under comparable representational conditions. The baseline and simple skip models overlap with the pulvinar model within this space but fail to achieve similar accuracy, whereas the SE model occupies a distinct region.

These results indicate that the observed effects cannot be explained by attention-like mechanisms, the addition of a parallel pathway, or optimization alone, but instead reflect a specific interaction between long-range routing and gain modulation. In this framework, the pulvinar does not primarily determine what is represented, but how representations are used to guide downstream processing.

This interpretation generates testable predictions: pulvinar perturbations should primarily affect gain regulation and decision criteria, with minimal impact on early sensory encoding, and effects that are strongest under uncertainty or when spatial integration is required. These effects should emerge preferentially at late, decision-related stages.

This perspective is consistent with recent evidence showing that neural signatures associated with conscious perception are more strongly related to late-stage, task-dependent activity than to early sensory encoding (Ferrante et al., 2025), suggesting that pulvinar-like modulation contributes to the conditions under which sensory representations become behaviorally effective.

### 4.4 Effects of pulvinar-like modulation on contrast detection near-threshold paradigm

The near-threshold contrast-detection task probed the effects of pulvinar-like modulation under high sensory uncertainty while minimizing demands on complex feature extraction. Consistent with this aim, pulvinar-inspired connectivity did not produce uniform improvements in accuracy or sensitivity. Instead, its effects emerged most clearly at the level of decision statistics.

This simplified stimulus regime was deliberately chosen to isolate the computational role of the transthalamic pathway under controlled conditions. By minimizing high-level feature complexity, the paradigm allows changes in gain regulation, representational stability, and decision criterion to be interpreted without confounds introduced by complex object structure or task-dependent strategies. Future work will be required to determine whether similar pulvinar-inspired control mechanisms operate in more naturalistic visual environments or in tasks requiring flexible switching between representations. Notably, a similar strategy has been used in studies of the mediodorsal (MD) thalamus, where simplified task structures have been employed to reveal how thalamocortical interactions support flexible routhing and reuse of cortical representation (Rikhye et al., 2018).

Across most stimulus configurations, pulvinar-like modulation redistributed detection outcomes rather than increasing sensitivity (d′) (Figures 5, 6). Under low-signal conditions, the pulvinar–cortical model showed increased false alarms and false negatives without proportional gains in hit rate, consistent with a liberal shift in decision criterion (Figures 6B, C). Such redistributions of detection outcomes without uniform gains in sensitivity are a hallmark of near-threshold perceptual decisions, in which internal decision criteria and evidence accumulation dynamics dominate performance (van Vugt et al., 2018). These effects were stimulus-dependent and confined to intrinsically difficult regimes, ruling out indiscriminate false detections (Summerfield and Tsetsos, 2015).

A distinct regime emerged when high background noise was combined with large stimulus size, where pulvinar-like connectivity supported genuine increases in sensitivity (Supplementary Figure 1 and Supplementary Table 2). These conditions place strong demands on spatial integration, suggesting that pulvinar modulation can enhance discriminability when global evidence accumulation is required (Gold, 2007). Outside this regime, pulvinar contributions were expressed primarily as criterion shifts.

This stimulus-dependent pattern suggests that pulvinar-like modulation does not exert a uniform influence on perceptual decisions but instead engages distinct computational regimes depending on the structure of sensory uncertainty. When background noise is high and target size is large, the observed improvement in sensitivity (d′) may reflect an increased ability to integrate information across spatially distributed inputs. In biological systems, the pulvinar has been proposed to facilitate coordination across cortical regions through synchronized activity, thereby enhancing the integration of distributed evidence (Saalmann et al., 2012; Cortes et al., 2020; Fiebelkorn et al., 2019). In this context, the gain in d′ may correspond to a network-level mechanism that promotes coherent integration of weak but spatially extended signals.

By contrast, under low-signal conditions with small targets, pulvinar-like modulation primarily induces shifts in decision criterion without substantial changes in sensitivity. This pattern is consistent with a more global regulation of decision thresholds under uncertainty, in which the system adjusts how evidence is weighted rather than improving the fidelity of sensory representations (Summerfield and Tsetsos, 2015; Feldman and Friston, 2010). Such behavior aligns with theoretical accounts of the pulvinar as a precision-control structure, modulating the effective impact of sensory inputs on downstream decision processes (Kanai et al., 2015; Cortes et al., 2024, 2026).

At the network level, these behavioral effects were mirrored by selective changes in late-stage activity. While early and intermediate layers showed comparable Hit–Miss separation across architectures, the most consistent differences arose in the PFC-like layer. Pulvinar-like modulation biased late-stage activity toward positive, decision-consistent states, consistent with the role of the prefrontal cortex in encoding decision variables rather than sensory representations (Heekeren et al., 2008).

### 4.5 Pulvinar-like modulation regulates precision and ignition thresholds

Contemporary theories propose that the pulvinar plays a key role in regulating cortical ignition—the transition from localized sensory processing to distributed, decision-relevant cortical states (Dehaene and Changeux, 2011; Cortes et al., 2024, 2026). Here, ignition is used in a functional sense, referring to the stabilization and large-scale recruitment of decision-relevant cortical states rather than subjective awareness. In these frameworks, ignition depends not only on evidence strength but on whether that evidence is assigned sufficient precision to engage large-scale cortical coordination, consistent with predictive processing accounts (Feldman and Friston, 2010; Kanai et al., 2015; Casanova and Chalupa, 2023). Importantly, recent large-scale adversarial tests of consciousness theories indicate that prefrontal signatures associated with conscious access predominantly reflect late, task-dependent decision states rather than enhanced sensory encoding *per se* (Ferrante et al., 2025).

In line with this view, pulvinar-like connectivity selectively reshaped late-stage cortical representations, biasing PFC activity toward decision-consistent states and shifting the internal decision criterion under uncertainty. These effects were most pronounced near the perceptual threshold, where inference is dominated by precision estimation rather than stimulus magnitude. From a mechanistic standpoint, this pattern supports an interpretation of the pulvinar as a precision-gating structure that modulates effective connectivity across cortical levels, facilitating ignition indirectly by shaping when weak or ambiguous evidence engages late-stage cortical dynamics (Cortes et al., 2024, 2026).

Importantly, this dual role—precision regulation and ignition facilitation—does not require the pulvinar to encode stimulus content explicitly. Rather, it positions the pulvinar as a control structure that stabilizes cortical dynamics by shaping when and how distributed cortical assemblies are recruited. Translating these principles into computational architectures suggests that pulvinar-inspired pathways should gate, stabilize, and contextually modulate interactions across levels, rather than merely shortcut hierarchical processing (Cortes and Vreeswijk, 2012, 2015). The present study provides an explicit test of this idea.

Recent experimental and computational studies of HO nuclei, particularly the MD thalamus, have shown that thalamocortical circuits regulate cortical gain, ensemble dynamics, and flexible routing of representations during cognitive tasks (Schmitt et al., 2017; Rikhye et al., 2018; Mukherjee et al., 2021; Wang B. A. et al., 2025; Zheng et al., 2024). Although these studies focus primarily on MD–prefrontal circuits, they support a broader view in which higher-order thalamic pathways modulate effective connectivity and the stability of cortical representations. Within the visual system, the pulvinar occupies an analogous anatomical position, forming extensive transthalamic loops with visual cortical areas. The computational effects observed in the present model (*i.e.*, contrast-dependent gain regulation, stabilization of representational geometry, and modulation of decision criteria under uncertainty), may therefore represent a visual-system instantiation of similar thalamocortical control principles.

Beyond these specific effects, the present results can be situated within a broader theoretical framework in which HO nuclei act as control structures over cortical computation. Recent work has emphasized that thalamocortical circuits contribute not only to routing of information, but also to its compression, regularization, and uncertainty-dependent modulation (Scott et al., 2024; Wang M. B. et al., 2025). Within this perspective, the pulvinar-like pathway implemented here can be interpreted as a concrete architectural instantiation of these principles: it regulates the influence of early sensory representations on downstream processing through a compressed summary of activity and a dynamic modulation of inter-layer interactions. This positions the pulvinar not as a feedforward relay or feature selector, but as a regulator of effective connectivity, shaping how information is integrated, stabilized, and deployed for decision-making under uncertainty. Consistent with work showing that HO circuits may act as integrative hubs coordinating cortical activity across perceptual and cognitive domains (*e.g.*, Halassa and Kastner, 2017; Shine, 2020), the results raise the possibility that similar computational motifs may generalize across sensory and cognitive domains.

## 5 Conclusion

Together, these results support a view of the pulvinar as a regulator of decision criteria and precision under uncertainty, rather than a direct amplifier of sensory signals. Pulvinar-like modulation shapes how evidence is accumulated and interpreted downstream, biasing late-stage representations toward stable decision states and selectively enhancing sensitivity only when global integration is required.

Importantly, our results further suggest that such effects arise from a core transthalamic mechanism of long-range routing and gain modulation, whose functional expression depends on the operating regime in which the network is embedded. This regime may be shaped by task demands and training constraints. This distinction parallel biological systems, where neuromodulatory constraints shape circuit dynamics without altering underlying connectivity.

This perspective provides a principled explanation for the context-dependent nature of pulvinar effects observed in both computational and neurophysiological studies.

## Data Availability

The raw data supporting the conclusions of this article will be made available by the authors, without undue reservation.
