# Supplementary material for: Pulvinar pathways as skip connections in deep neural networks for vision
Source: Front Neuroimaging. 2026 May 14;5:1800369. doi: 10.3389/fnimg.2026.1800369 (PMC13215803; doi:10.3389/fnimg.2026.1800369)
Supplement: Supplementary file 1 [file Data_Sheet_1.pdf]

# *Supplementary Material*

**Supplementary Table 1. Mean absolute counts of signal-detection outcomes (TP, FN, FP, TN) across paired cortical-only and pulvinar-augmented simulations, reported globally and by stimulus quadrant (Q1–Q4).** Differences (Δ = Pulvinar − Cortex) reflect redistributions of decision outcomes rather than normalized performance changes. Rate-based metrics (H, FA) are reported separately.

| **Metric** | **Region** | **n (paired)** | **Mean Cortex** | **Mean Pulvinar** | **Mean Δ (P−C)** | **Test** | **p-value** |
|---|---|---|---|---|---|---|---|
| **TP** | GLOBAL | 180 | 2298 | 2239 | −59.6 | two-sided | **0.00071** |
| TP | Q1 | 48 | 2313 | 2234 | −78.7 | two-sided | 0.101 |
| TP | Q2 | 48 | 2243 | 2256 | 13 | two-sided | 0.306 |
| **TP** | **Q3** | 42 | 2326 | 2157 | −169.1 | two-sided | **0.0000239** |
| TP | Q4 | 42 | 2316 | 2305 | −11.2 | two-sided | 0.105 |
| **FN** | GLOBAL | 180 | 203.9 | 263.4 | 59.6 | greater | **0.000358** |

| | | | | | | | |
|---|---|---|---|---|---|---|---|
| FN | Q1 | 48 | 189.8 | 268.5 | 78.7 | greater | 0.05 |
| FN | Q2 | 48 | 262.4 | 249.4 | −13.0 | greater | 0.851 |
| **FN** | **Q3** | 42 | 171.9 | 341 | 169.1 | greater | **0.000012** |
| FN | Q4 | 42 | 185 | 196.1 | 11.2 | greater | 0.052 |
| FP | GLOBAL | 180 | 682.9 | 742.4 | 59.5 | greater | 0.398 |
| **FP** | **Q1** | 48 | 1364 | 1599 | 235.4 | greater | **0.026** |
| FP | Q2 | 48 | 398.2 | 337.6 | −60.6 | greater | 0.883 |
| FP | Q3 | 42 | 698.5 | 827.2 | 128.7 | greater | 0.113 |
| FP | Q4 | 42 | 214.6 | 141.3 | −73.3 | greater | 0.996 |
| TN | GLOBAL | 180 | 1815 | 1756 | −59.5 | two-sided | 0.797 |
| TN | Q1 | 48 | 1134 | 898.7 | −235.4 | two-sided | 0.053 |
| TN | Q2 | 48 | 2096 | 2157 | 60.6 | two-sided | 0.239 |

| | | | | | | | |
|---|---|---|---|---|---|---|---|
| TN | Q3 | 42 | 1803 | 1674 | −128.7 | two-sided | 0.226 |
| **TN** | **Q4** | 42 | 2285 | 2358 | 73.3 | two-sided | **0.0077** |
| **H** | GLOBAL | 180 | 0.9185 | 0.8946 | −0.0239 | two-sided | **0.00071** |
| **H** | **Q3** | 42 | 0.9311 | 0.8632 | −0.0679 | two-sided | **0.0000223** |
| FA | GLOBAL | 180 | 0.2736 | 0.2973 | 0.0237 | greater | 0.394 |
| **FA** | **Q1** | 48 | 0.5466 | 0.6405 | 0.0938 | greater | **0.026** |

**Supplementary Table 2. Frequency of high-valued signal-detection events in cortical-only (Cortex) and pulvinar–cortical (Pulvinar) models.** For each metric (TP, FN, FP, TN, H), values indicate the number of stimulus conditions classified as high (high / total), reported globally and by stimulus quadrant (Q1–Q4), with corresponding percentages. Quadrants reflect combinations of target size (R) and background noise (BG).

| **Metric** | **Region** | **Cortex (high / total)** | **Cortex frac** | **Pulvinar (high / total)** | **Pulvinar frac** |
|---|---|---|---|---|---|
| TP | GLOBAL | 17 / 180 | 9.40% | 12 / 180 | 6.70% |
| TP | Q1 | 13 / 48 | 27.10% | 10 / 48 | 20.80% |
| TP | Q2 | 0 / 48 | 0.00% | 0 / 48 | 0.00% |

| | | | | | |
|---|---|---|---|---|---|
| TP | Q3 | 4 / 42 | 9.50% | 2 / 42 | 4.80% |
| TP | Q4 | 0 / 42 | 0.00% | 0 / 42 | 0.00% |
| FN | GLOBAL | 16 / 180 | 8.90% | 22 / 180 | 12.20% |
| FN | Q1 | 6 / 48 | 12.50% | 9 / 48 | 18.80% |
| FN | Q2 | 6 / 48 | 12.50% | 0 / 48 | 0.00% |
| FN | Q3 | 2 / 42 | 4.80% | 13 / 42 | 31.00% |
| FN | Q4 | 2 / 42 | 4.80% | 0 / 42 | 0.00% |
| FP | GLOBAL | 30 / 180 | 16.70% | 38 / 180 | 21.10% |
| FP | Q1 | 22 / 48 | 45.80% | 30 / 48 | 62.50% |
| FP | Q2 | 0 / 48 | 0.00% | 0 / 48 | 0.00% |
| FP | Q3 | 8 / 42 | 19.00% | 8 / 42 | 19.00% |
| FP | Q4 | 0 / 42 | 0.00% | 0 / 42 | 0.00% |
| TN | ALL REGIONS | 0 / * | 0.00% | 0 / * | 0.00% |

| | | | | | |
|---|---|---|---|---|---|
| H | GLOBAL | 15 / 180 | 8.30% | 12 / 180 | 6.70% |
| H | Q1 | 12 / 48 | 25.00% | 10 / 48 | 20.80% |
| H | Q2 | 3 / 42 | 7.10% | 2 / 42 | 4.80% |

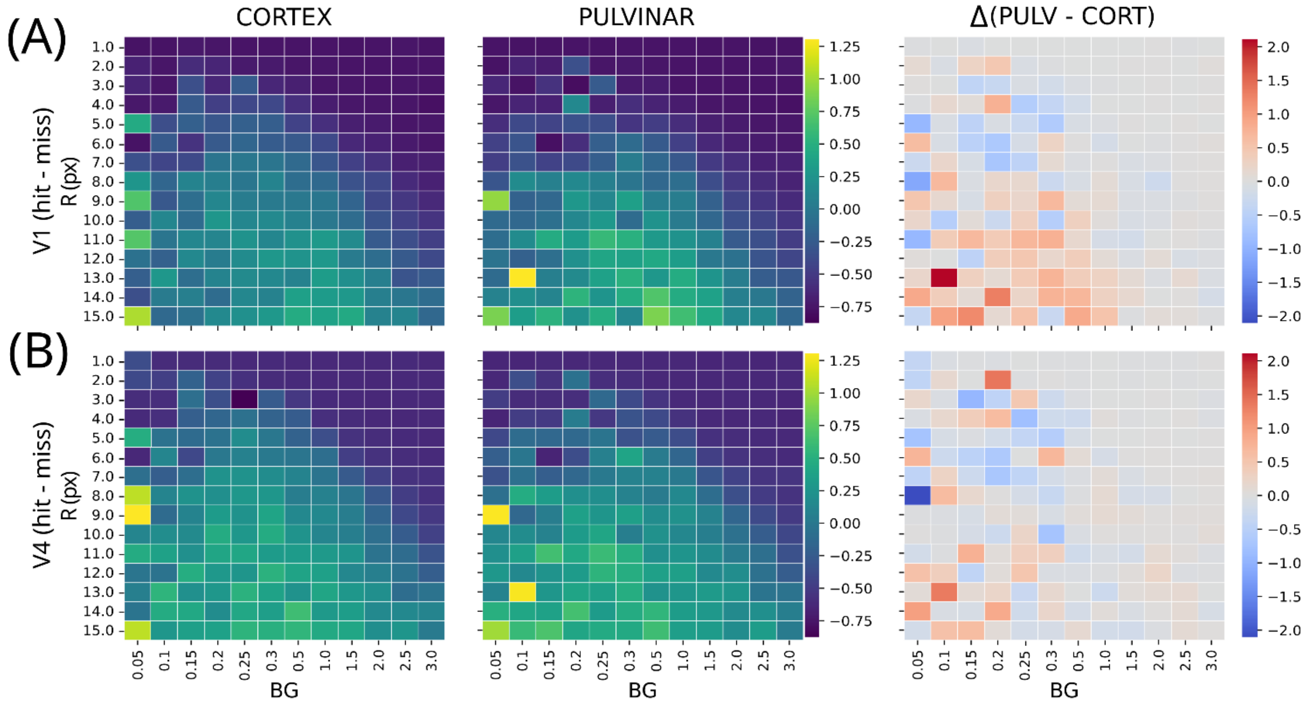


**Supplementary Figure 1.** Global activation of V1 and V4 for networks as a function of target radius (R) and background noise (BG). Left: cortical-only model. Middle: pulvinar–cortical model. Right: paired difference (pulvinar − cortex).

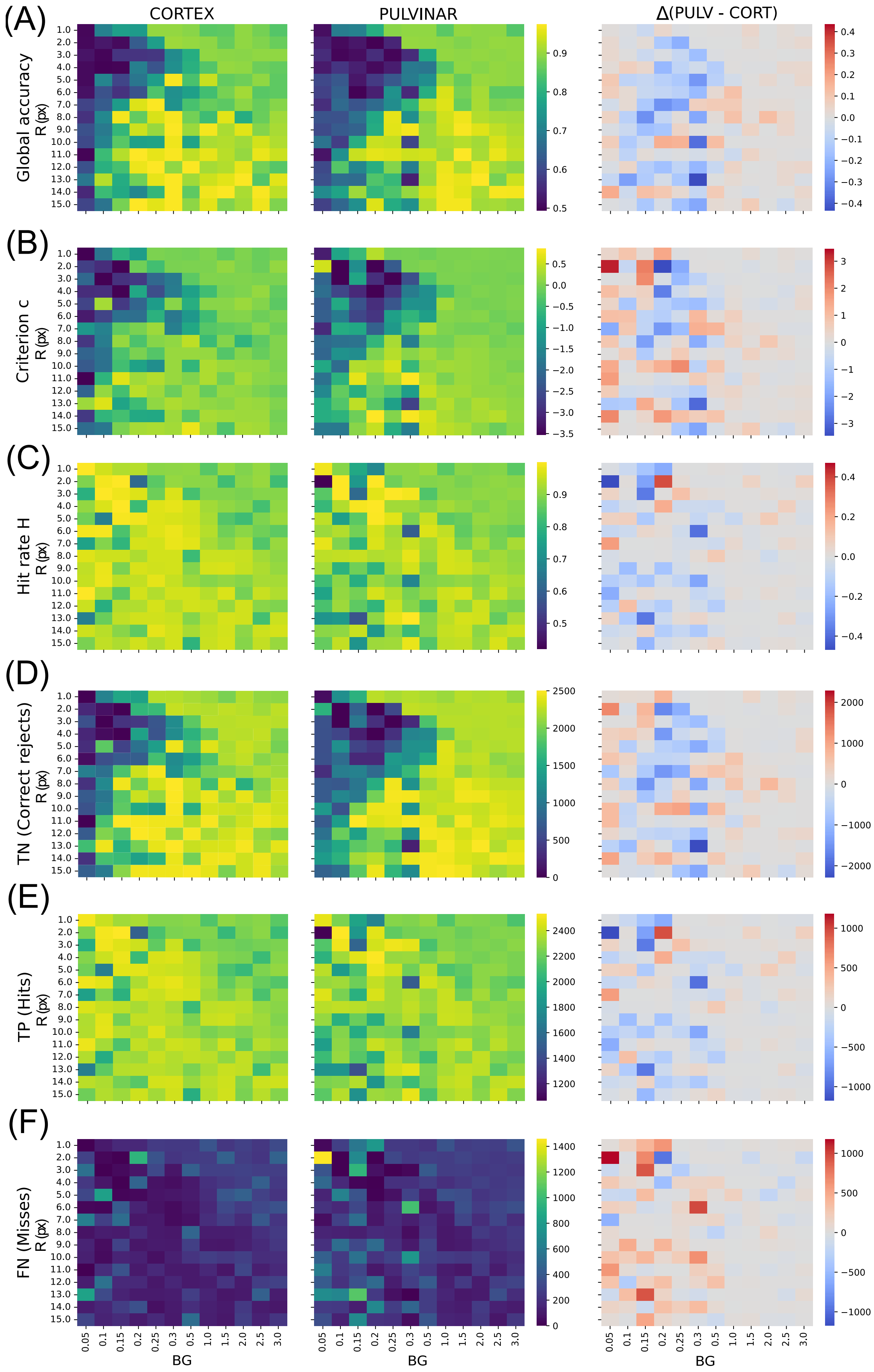

(A)
CORTEX
PULVINAR
Δ(PULV - CORT)
Global accuracy
R (px)
(B)
Criterion c
R (px)
(C)
Hit rate H
R (px)
(D)
TN (Correct rejects)
R (px)
(E)
TP (Hits)
R (px)
(F)
FN (Misses)
R (px)
BG

**Supplementary Figure 2.** Spatial heatmaps of network activity across task conditions.
Each row (A–F) corresponds to decision outcomes: A) Accuracy, B) Decision criterion, C) Sensitivity ($d'$), D) Hit rate, E) False-alarm rate, and F) FN (misses). Columns show the cortical-only network (left), the cortical–pulvinar network (middle), and the difference map (right; pulvinar − cortical). Heatmaps depict spatially resolved activity values on a common grid. Cortical-only and cortical–pulvinar panels use identical color scales within each column, while difference maps use a zero-centered diverging colormap, with warm colors indicating increased activity and cool colors indicating decreased activity due to pulvinar modulation.
