# Supplementary material for: Pulvinar pathways as skip connections in deep neural networks for vision
Source: Front Neuroimaging. 2026 May 14;5:1800369. doi: 10.3389/fnimg.2026.1800369 (PMC13215803; doi:10.3389/fnimg.2026.1800369)
Supplement: Supplementary file 2 [file Data_Sheet_2.docx]

**Supplementary Table 3.** Accuracy (Dunn post-hoc, Holm-corrected p-values). models.

|  | Cortex | Cortex + reg | Pulvinar | Pulvinar + reg | Skip | Skip + reg | SE | SE + reg |
| --- | --- | --- | --- | --- | --- | --- | --- | --- |
| Cortex | 1 | 0 | 0 | 0 | 1 | 0 | 1 | 0 |
| Cortex + reg | 0 | 1 | 1 | 0 | 0 | 1 | 0 | 1 |
| Pulvinar | 0 | 1 | 1 | 0 | 0 | 0.231 | 0 | 0.471 |
| Pulvinar + reg | 0 | 0 | 0 | 1 | 0 | 0 | 0 | 0 |
| Skip | 1 | 0 | 0 | 0 | 1 | 0 | 1 | 0 |
| Skip + reg | 0 | 1 | 0.231 | 0 | 0 | 1 | 0 | 1 |
| SE | 1 | 0 | 0 | 0 | 1 | 0 | 1 | 0 |
| SE + reg | 0 | 1 | 0.471 | 0 | 0 | 1 | 0 | 1 |

**Supplementary Table 4.** Directional invariance (S) (Dunn post-hoc, Holm-corrected p-values).

|  | Cortex | Cortex + reg | Pulvinar | Pulvinar + reg | Skip | Skip + reg | SE | SE + reg |
| --- | --- | --- | --- | --- | --- | --- | --- | --- |
| Cortex | 1 | 0 | 1 | 0 | 0.006 | 0 | 0.021 | 0 |
| Cortex + reg | 0 | 1 | 0 | 1 | 0 | 0 | 0 | 0 |
| Pulvinar | 1 | 0 | 1 | 0 | 0 | 0 | 0.001 | 0 |
| Pulvinar + reg | 0 | 1 | 0 | 1 | 0 | 0 | 0 | 0.001 |
| Skip | 0.006 | 0 | 0 | 0 | 1 | 0 | 1 | 0 |
| Skip + reg | 0 | 0 | 0 | 0 | 0 | 1 | 0 | 1 |
| SE | 0.021 | 0 | 0.001 | 0 | 1 | 0 | 1 | 0 |
| SE + reg | 0 | 0 | 0 | 0.001 | 0 | 1 | 0 | 1 |

**Supplementary Table 5.**  Gain linearity (HL) (Dunn post-hoc, Holm-corrected p-values).

|  | Cortex | Cortex + reg | Pulvinar | Pulvinar + reg | Skip | Skip + reg | SE | SE + reg |
| --- | --- | --- | --- | --- | --- | --- | --- | --- |
| Cortex | 1 | 0 | 1 | 0 | 0 | 0 | 0.052 | 0 |
| Cortex + reg | 0 | 1 | 0 | 0.668 | 0 | 0 | 0 | 0 |
| Pulvinar | 1 | 0 | 1 | 0 | 0 | 0 | 0.009 | 0 |
| Pulvinar + reg | 0 | 0.668 | 0 | 1 | 0 | 0.001 | 0 | 0.001 |
| Skip | 0 | 0 | 0 | 0 | 1 | 0 | 0.162 | 0 |
| Skip + reg | 0 | 0 | 0 | 0.001 | 0 | 1 | 0 | 1 |
| SE | 0.052 | 0 | 0.009 | 0 | 0.162 | 0 | 1 | 0 |
| SE + reg | 0 | 0 | 0 | 0.001 | 0 | 1 | 0 | 1 |
